# Clinical effectiveness of paliperidone palmitate 3‐monthly and 1‐monthly as monotherapy in patients with schizophrenia: A retrospective cohort study based on the Medicaid claims database

**DOI:** 10.1002/npr2.12473

**Published:** 2024-09-11

**Authors:** Chih‐Lin Chiang, Madoka Chinen, Mehmet Daskiran, Akihide Wakamatsu, Ibrahim Turkoz

**Affiliations:** ^1^ Medical Affairs, Johnson & Johnson Innovative Medicine Taipei Taiwan; ^2^ Medical Affairs, Janssen Pharmaceutical K.K. Tokyo Japan; ^3^ US Statistics & Decision Sciences, Janssen Research & Development, LLC Titusville New Jersey USA

**Keywords:** adherence, monotherapy, paliperidone palmitate, relapse, schizophrenia

## Abstract

**Aim:**

Real‐world data (RWD) for paliperidone palmitate (PP) three‐monthly (PP3M) is lacking based on Japan label requirements. This study evaluated the clinical effectiveness of PP3M versus PP once‐monthly (PP1M) in patients with schizophrenia administered according to Japan label requirements.

**Methods:**

Retrospective analyses were conducted using RWD from Merative™ MarketScan® Multi‐State Medicaid (MDCD) claims database (June 2015–December 2022). Adult patients with schizophrenia switching from PP1M to PP3M were included. Patients transitioning to PP3M were matched with patients who continued with PP1M using propensity score matching (PSM) at 1:1 ratio. Primary hypothesis aimed to investigate non‐inferiority of PP3M versus PP1M in terms of relapse‐free status at 24 months from index PP injection. Outcome measures were proportions of relapse‐free patients at 24 months, time to relapse, treatment persistence, and adherence.

**Results:**

Total 4252 eligible adult schizophrenia patients on PP (PP3M:582; PP1M:3670) were identified. After PSM, each PP cohort comprised 562 matched individuals. Estimated proportion of relapse‐free patients was higher in PP3M (85.7%) versus PP1M (77.9%), per Japan PP label. PP3M demonstrated superiority to PP1M after testing for non‐inferiority in terms of achieving relapse‐free status at 24 months, with an estimated difference of 7.8% (95% CI: 1.7%–13.9%). PP3M cohort had lower risk of relapse (HR: 0.605; CI: 0.427–0.856), longer treatment persistence, and higher treatment adherence versus PP1M cohort.

**Conclusions:**

Findings suggests that patients who switched to PP3M might be able to reduce risk of relapse compared to those who continued PP1M after aligning particularly with Japan's label requirements.

## Abstract 2

Japan's label requirements for PP3M prohibit the concurrent use of antipsychotic medication during the transition to PP3M and after initiating PP3M treatment. Analysis using the Merative™ MarketScan® Multi‐State Medicaid claims database revealed that adult patients with schizophrenia who switched to PP3M had a significantly lower risk of relapse compared to those who remained on PP1M. The risk difference at 24 months was 7.8% (95% CI: 1.7–13.9), with a hazard ratio (HR) of 0.605 (95% CI: 1.7–13.9), when using analytical methods consistent with Japan's label.

## Graphical abstract

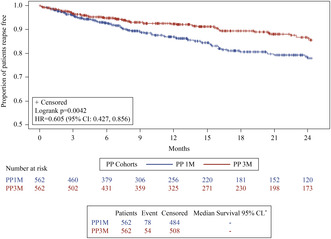

## 1 INTRODUCTION

Schizophrenia is a chronic psychiatric condition often characterized by recurrent episodes of psychosis, which can cause significant impairment in social and occupational functioning.^1^ The condition is commonly manifested as hallucinations, delusions, and diminished cognition and perception.^2^ According to the Global Burden of Diseases report, the global prevalence of schizophrenia (14.2–23.6 million) and disability‐adjusted life years (9.1–15.1 million) increased by more than 65% and 65%, respectively, between 1990 and 2019.^3^ In 2019, the estimated lifetime prevalence of schizophrenia among the Japanese population was 0.59% (95% CI: 0.51%–0.68%).^4^

The recurrence of symptoms of schizophrenia or relapse is linked to various detrimental consequences, including violence, strained relationships, increased social stigma, disruptions in employment and education, reduced brain volume, worsening of baseline functioning, increased economic and healthcare burden, and overall mortality.^5^, ^6^, ^7^, ^8^ Many individuals with schizophrenia in Japan suffer frequent symptomatic exacerbations and relapses, often triggered due to non‐adherence or discontinuation of treatment.^9^, ^10^ Thus, the primary objective of schizophrenia management is improving adherence and preventing relapse.^11^ Treatment with antipsychotic medications can induce symptomatic remissions, prevent relapses, and reduce the risk of death in patients with schizophrenia.^7^, ^12^

Compared to oral antipsychotics, long‐acting injectable antipsychotics (LAI) eliminate the need for daily dosing^13^ and have shown to achieve lower the risk of relapse and hospitalization.^14^, ^15^, ^16^, ^17^, ^18^, ^19^, ^20^ Second‐generation antipsychotic paliperidone palmitate (PP) comes in once‐monthly (PP1M) and three‐monthly (PP3M) dosage formulations.^21^ The US Food and Drug Administration (August 31, 2021) and the European Medicines Agency (November 23, 2021), recently approved PP 6‐monthly formulation (PP6M) and it has shown to be non‐inferior to PP3M in preventing relapse.^22^, ^23^ However, only PP1M and PP3M are currently available in Japan. In terms of lowering the likelihood of relapsing into schizophrenia, PP3M has demonstrated effectiveness over placebo^24^ and non‐inferiority versus PP1M.^25^ Furthermore, a retrospective analysis utilizing information from the US Medicaid claims database reported that the risk of relapse was significantly lower and treatment adherence was significantly higher in the PP3M group compared to the PP1M group.^26^ These results imply that LAIs, and in particular the 3‐monthly formulation of PP, might be a useful therapeutic alternative for avoiding schizophrenia relapses.

The use of antipsychotic polypharmacy in patients with schizophrenia, with or without LAI as part of the antipsychotic regimen, is a topic of ongoing debate.^27^, ^28^, ^29^ Many clinical guidelines have not recommended its use due to concerns regarding safety, while other has no such restrictions. In this sense, there are marked differences between the Japan versus US PP3M package insert (PI).

According to Japan PP3M PI, the approved patient population for PP3M is limited to patients with schizophrenia who have been stabilized with PP1M monotherapy^30^, ^31^ for at least 4 months. These patients must not have used any concomitant antipsychotics (including oral paliperidone/risperidone) and must have received the last two dose of PP1M at the same dose strength to confirm their stable clinical condition before switching to PP3M. Post‐switching, patients must continue with PP3M monotherapy without concomitant antipsychotics and must switch back to PP1M if dose changes are necessary.^32^ In contrast, the US PI does not impose conditions on the use of concomitant antipsychotics during PP3M treatment. This implies that in United States, patients can be on PP3M treatment without much restrictions.^33^

The result of a recently completed clinical trial showed non‐inferiority of PP3M to PP1M.^25^ However, it is unclear whether PP3M is as effective as PP1M in unique Japan label context, even though several retrospective database studies in other countries have demonstrated the clinical effectiveness of PP3M in real‐world (RW) settings.^26^, ^34^, ^35^ Therefore, we conducted this study to demonstrate the non‐inferiority of PP3M compared to PP1M in maintaining relapse‐free status in Medicaid patients with schizophrenia using health claims data generated in routine clinical practice. Superiority of PP3M over PP1M was also evaluated to confirm the hypothesis of no difference. Due to the limited number of PP3M‐treated patients in the Japan's claims databases, we chose to use the US‐based Merative™ MarketScan® Multi‐State Medicaid (MDCD) database for our analysis.

## 2 METHODS

### 2.1 Data sources

This study utilized data from the MDCD, a comprehensive US claims database. The MDCD is a database of healthcare coverage and service use for individuals enrolled in State Medicaid programs and/or Medicaid managed care programs in the United States. The database includes details such as enrollment information (demographics, enrollment periods, and plan types), records of inpatient and outpatient services, medical and pharmacy claims and financial data of more than 30 million Medicaid enrollees from around 10 states.

The use of MDCD was assessed and exempted from review board approval by the New England Institutional Review Board, as it does not involve research in human subjects. All data were de‐identified and fully compliant with the regulations of the US Health Insurance Portability and Accountability Act of 1996.

International Classification of Diseases, Ninth Revision, Clinical Modification (ICD‐9‐CM), International Classification of Diseases, Tenth Revision, Clinical Modification (ICD‐10‐CM), Diagnosis Related Group (DRG), National Drug Code, Current Procedural Terminology, and The Healthcare Common Procedure Coding System codes were used to retrieve data related to diagnoses, medication records, and procedures (Supplementary Table 1).

### 2.2 Study design and patients

**FIGURE 1:**
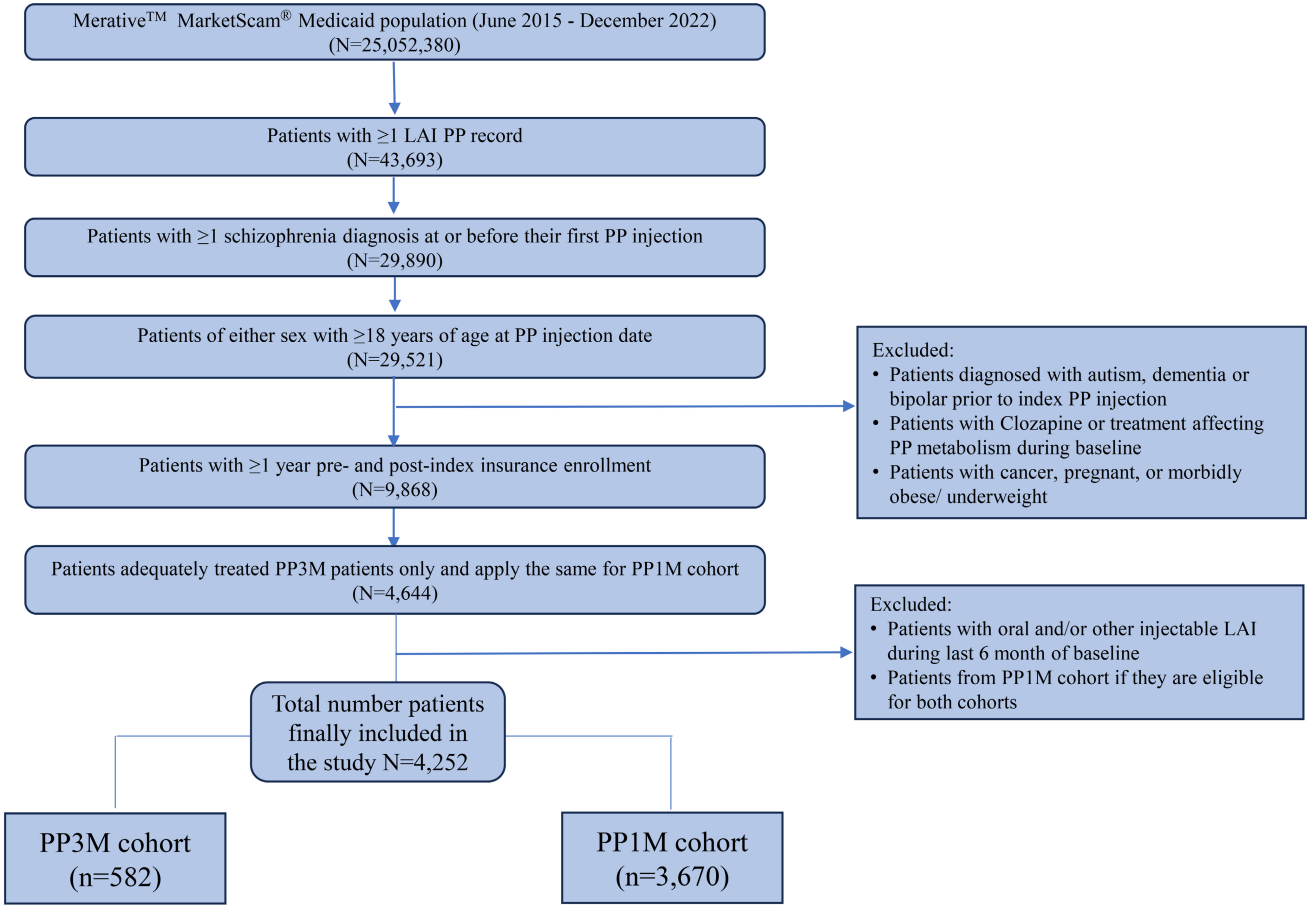
Patient attrition summary. LAI, long‐acting injectable; PP, paliperidone palmitate; PP1M, paliperidone palmitate 1‐monthly; PP3M, paliperidone palmitate 3‐monthly.

This retrospective propensity score matching (PSM) cohort study was conducted from June 2015 to December 2022, comparing two patient cohorts: PP3M and PP1M. Date of the first eligible PP3M or PP1M drug claims prescription record during study period were assigned as the index date. The primary objective of this study was to demonstrate the non‐inferiority of PP3M compared to PP1M in maintaining relapse‐free status in patients with schizophrenia. Secondary objective was to demonstrate that PP3M is more effective than PP1M in maintaining relapse‐free status in patients with schizophrenia after testing for non‐inferiority. Other secondary objectives were to describe the treatment persistence and adherence of LAI PP during study period in patients with schizophrenia. Patients were required to have both continuous insurance and prescription drug coverage and followed up from the index date until they exit from the insurance plan (excluding breaks of <31 days), or up to 2 year after the study index date, or the end of the available data in the database, whichever occurred first. Only patients with Medicaid coverage records were eligible and those with dual eligibility (Medicaid and Medicare) were excluded. Other exclusion criteria included: patients with other mental conditions (autism, dementia, and mania/bipolar) at the index date or during the 12‐month baseline period; patients treated with clozapine at index date or during the 2 months before the index date or during the 6‐month stabilization period; patients treated with a concomitant antipsychotics (other than risperidone/paliperidone (any formulation), or risperidone/paliperidone of a non‐LAI formulation) on the index date; patients treated with only 39 mg of PP1M (since there is no corresponding equivalent PP3M strength); patients with concomitant treatment of a strong CYP3A4 inducer (e.g., carbamazepine, which affects PP metabolism) at the index date or during the 5 months before the index date (Figure 1).

PP3M patients were evaluated first and eligible PP3M patients were removed from the PP1M pool prior to identifying eligible PP1M population. Patients of either sex aged 18 years and older who had at least one claim for schizophrenia diagnosis, anytime up to the index visit, and a continuous enrollment in the insurance and prescription drug plan for at least 12 months prior to the index date (i.e., baseline period) and 12 months after the index date (i.e., follow‐up period) were included in this study. The maximum follow‐up period was set to 2 years. For PP3M cohort, patients stable at PP3M injection date (at least five PP1M injections prior to index PP3M injection date with the last two PP1M strength being same) and for PP1M cohort, patients stable at PP1M injection date (at least five PP1M injections prior to PP1M injection date and last two PP1M strength being same) were included in PSM process.

### 2.3 Propensity score matching

All eligible patients from the MDCD database treated with PP3M and PP1M and met the selection criteria were included in the matching process. Patients in the PP3M and PP1M cohorts were assigned into one of four drug dose categories (Supplementary Table 2) based on their PP formulation and injection dose at study entry (index date). Propensity scores were calculated using the following factors: (i) exact match categories included index PP injection year, index PP drug dose, age category at index, gender and (ii) non‐exact matching factors included race, presence of baseline depressive disorder, relapse events and mental health‐related inpatient hospitalizations at baseline, and Charlson's Comorbidity Index (CCI) Score and Elixhauser Comorbidity Index Score domains calculated using medical records during baseline period. In the primary analysis, PP3M‐ and PP1M‐treated patients were matched at a 1:1 ratio using the nearest neighbor matching algorithm with a caliper of 0.2 standard deviation (SD) without replacement so that index drug dose, age category, gender, and index year were an exact match. The quality of the PSM was assessed using the standardized mean differences (SMD) for each baseline factor. The SMD is a measure of the difference in mean values between the two groups being compared, adjusted for the variance in the data. Before and after matching, SMDs were computed and compared. An SMD < 0.10 was considered a good match, and an SMD up to 0.20 was considered acceptable.^36^

### 2.4 Outcomes and analysis

The primary outcome measure was the difference in 2‐year relapse‐free rate based on Kaplan–Meier estimate of survival (i.e., proportion of patients who remained relapse free at month‐24). Relapse was determined to occur if a patient had a claims record for any of the following: inpatient hospitalization with a primary mental health‐related diagnosis (ICD‐9‐CM, ICD‐10‐CM) or DRG code, suicidal behavior, suicide attempt, injury with undetermined intent, suicidal ideation, homicidal ideation, exacerbation of schizophrenia, clozapine use, violent behavior, hostility, and aggressive behavior. The time to relapse was considered as the day of first occurrence of any of the events in these relapse criteria during the follow‐up period. Additionally, a separate analysis was conducted considering the Japan label restrictions of PP3M.^30^ Per Japan PP3M label, if a direct change of the PP3M dosage or the introduction of concomitant antipsychotic use is clinically required, the patient should be switched back to PP1M for restabilization.^30^ In this analysis, a patient was also censored at the first occurrence of switch in index PP formulation or change in index PP dose or the initiation of concomitant antipsychotic and relapse events occurring after this date were not included in analysis.

The secondary outcome measures included (a) duration of persistence in LAI PP treatment and (b) adherence of LAI PP treatment. Persistence was determined by calculating the duration of continuous medication supply, considering that a PP1M injection covers 28 days, a PP3M injection covers 84 days, and a grace period of 14 days for PP1M and 28 days for PP3M are allowed for any gaps between refills. Adherence was measured using the proportion of days covered (PDC), defined as the number of days of the study period covered by a PP3M or PP1M prescription divided by the total number of days in follow‐up period.

### 2.5 Statistical analysis

A feasibility assessment based on the MDCD revealed that as of June 2021, there were approximately 500 patients treated with PP3M and 2200 patients treated with PP1M who were eligible for the PSM based on the selection criteria. Based on the results of the feasibility assessment, the primary analysis required 400 or more eligible PP patients in each cohort (PP3M and PP1M) after PSM. This sample size was deemed sufficient to provide adequate power for the study. The sample size determination included the assumptions that the expected survival rate (percentage of patients who remained relapse free at 24 months) in the PP1M group was assumed to be 75%, and that the one‐sided significance level should be 2.5%. Given these assumptions, approximately 400 patients per group in a 1:1 ratio (PP3M:PP1M) were required to demonstrate with 90% power that PP3M is no worse than PP1M by a non‐inferiority margin of 10% for the percentage of patients remaining relapse free at 24 months.

Descriptive statistics was used to summarize the patient characteristics and outcome measures for each cohort. Kaplan–Meier method was used to estimate the 2‐year cumulative estimate of survival (i.e., proportion of patients who remained relapse free). Non‐inferiority of PP3M to PP1M was concluded if the lower limit of the two‐sided 95% (one‐sided alpha = 0.025) CI of the difference in relapse‐free rates between PP3M and PP1M exceeded the preselected non‐inferiority margin of −10%. In the previous non‐inferiority trials involving PP3M, the preselected non‐inferiority margin for the relapse‐free rate at 12 months was set at −10%^23^ or −15%.^25^ In the case of non‐inferiority, it is acceptable to calculate the *p*‐value associated with a test of superiority and to evaluate whether this is sufficiently small to reject convincingly the hypothesis of no difference. There is no multiplicity argument that affects this interpretation because, in statistical terms, it corresponds to a simple closed test procedure. Additionally, treatment differences were compared using a log‐rank test. Cox model was used to calculate the hazard ratio (HR) and 95% CI to describe the reduction in risk of relapses. Persistency and adherence were evaluated using a two‐sample paired t‐test. Additional analyses based on PSM ratio of (1:2) for PP3M‐ and PP1M‐treated patients were also performed to assess the robustness of the results.

## 3 RESULTS

### 3.1 Patient disposition and demographics

**FIGURE 2:**
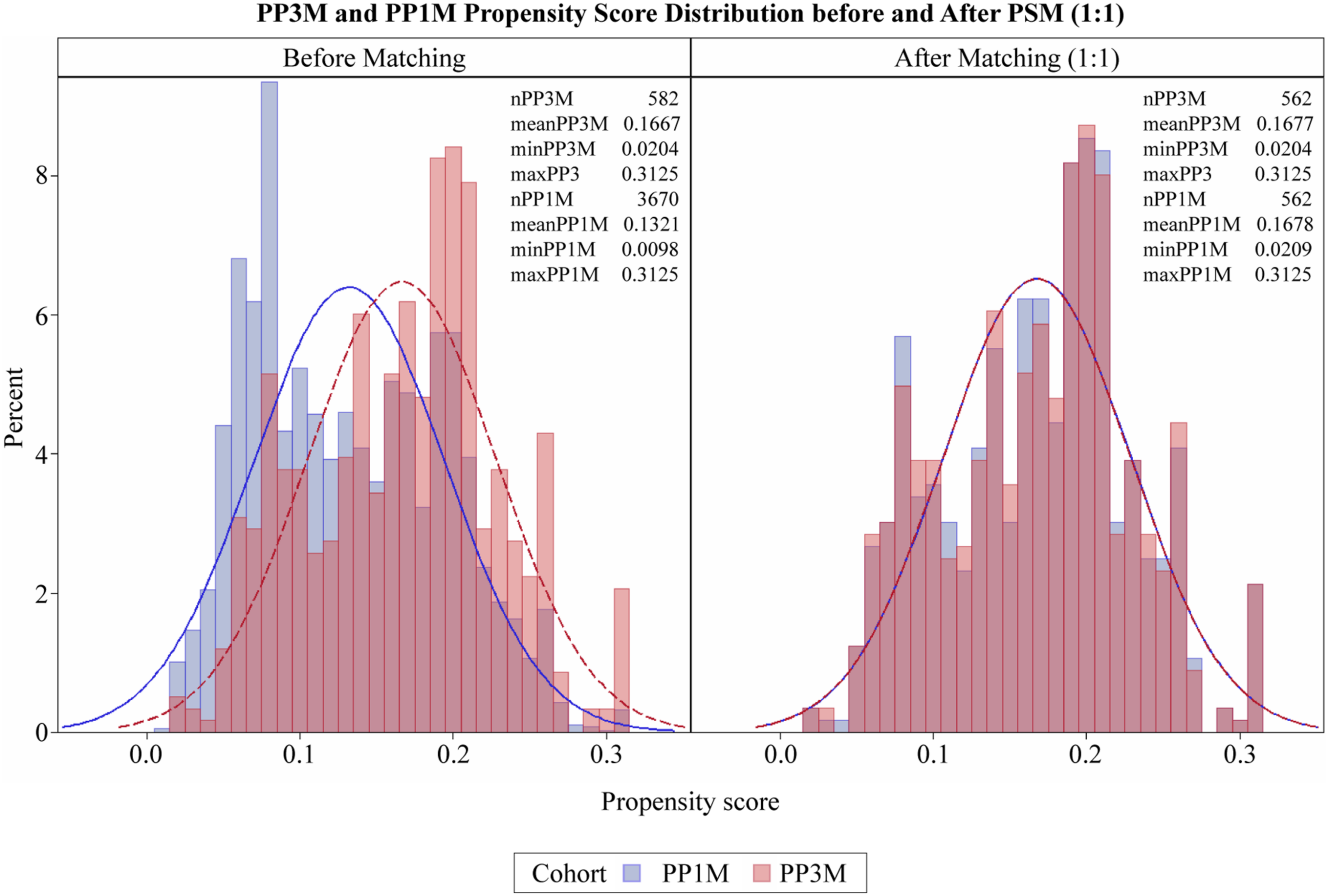
PP3M and PP1M propensity score distribution before and after PSM (1:1). PP1M, paliperidone palmitate 1‐monthly; PP3M, paliperidone palmitate 3‐monthly; PSM, propensity score matching.

Of the total 4252 patients included from the MDCD, PP1M cohort consisted of 3670 patients and PP3M cohort included 582 patients as shown in Figure 1 and Supplementary Table 3. After PSM at 1:1 ratio as shown in Figure 2, a total 562 patients (female 22.1%, mean age: 37.7 years for PP3M, 37.4 years for PP1M) were matched and included in each cohort (Table 1). Most of the baseline characteristics including baseline comorbidities, depressive disorder, prior history of relapse event, and prior mental health‐related inpatient hospitalization were similar in both cohorts. The percentage of patients with prior mental health emergency room usage was lower in the PP3M cohort (11.6%) compared to PP1M cohort (15.7%). Most of the patients were taking the highest dose of PP (Table 1). Elixhauser and CCI domains with their SMDs are presented in Supplementary Table 4. Most patient characteristics after PSM had SMDs < 0.1 and were well balanced.

**TABLE 1:** Baseline demographics and disease state characteristics for PP3M and PP1M cohorts before and after PSM at ratio 1:1.

Patient characteristics	Before matching			After matching		
	PP3M	PP1M	SMD	PP3M	PP1M	SMD
	*n* = 582	*n* = 3670		*n* = 562	*n* = 562	
Age, mean (SD), years	37.8 (12.14)	38.6 (12.83)	0.067	37.7 (12.07)	37.4 (12.27)	0.028
Age category, *n* (%)						
18–25 years	96 (16.5)	583 (15.9)	0.017	90 (16.0)	90 (16.0)	0.000
26–50 years	378 (64.9)	2224 (60.6)	0.090	370 (65.8)	370 (65.8)	0.000
≥51 years	108 (18.6)	863 (23.5)	0.122	102 (18.1)	102 (18.1)	0.000
Sex (female), *n* (%)	132 (22.7)	990 (27.0)	0.100	124 (22.1)	124 (22.1)	0.000
Race/ethnicity, *n* (%)						
Black	320 (55.0)	1883 (51.3)	0.074	310 (55.2)	330 (58.7)	0.072
White	131 (22.5)	867 (23.6)	0.026	130 (23.1)	127 (22.6)	0.013
Mixed/unknown	74 (12.7)	468 (12.8)	0.001	71 (12.6)	58 (10.3)	0.073
Other	37 (6.4)	312 (8.5)	0.082	31 (5.5)	36 (6.4)	0.038
Hispanic	9 (1.5)	49 (1.3)	0.018	9 (1.6)	2 (0.4)	0.127
Missing	11 (1.9)	91 (2.5)	0.040	11 (2.0)	9 (1.6)	0.027
CCI score, mean (SD)	0.5 (1.11)	0.6 (1.17)	0.094	0.4 (1.10)	0.4 (0.94)	0.057
Elixhauser Comorbidity Index score, mean (SD)	2.5 (1.79)	3.0 (1.99)	0.228	2.5 (1.77)	2.5 (1.54)	0.032
Depressive disorder diagnosis, *n* (%)	105 (18.0)	960 (26.2)	0.197	99 (17.6)	98 (17.4)	0.005
Index PP dose, *n* (%)						
175/50 mg eq.	8 (1.4)	54 (1.5)	0.008	3 (0.5)	3 (0.5)	0.000
263/75 mg eq.	99 (17.0)	522 (14.2)	0.077	94 (16.7)	94 (16.7)	0.000
350/100 mg eq.	228 (39.2)	1545 (42.1)	0.060	224 (39.9)	224 (39.9)	0.000
525/150 mg eq.	247 (42.4)	1549 (42.2)	0.005	241 (42.9)	241 (42.9)	0.000
Prior relapse event^a^, *n* (%)	97 (16.7)	1105 (30.1)	0.322	91 (16.2)	90 (16.0)	0.005
Prior mental health inpatient hospitalization, *n* (%)	82 (14.1)	961 (26.2)	0.305	76 (13.5)	78 (13.9)	0.010
Prior mental health ER visit, *n* (%)	67 (11.5)	723 (19.7)	0.227	65 (11.6)	88 (15.7)	0.120

Abbreviations: CCI, Charlson's Comorbidity Index; eq., equivalent; ER, emergency room; PP, paliperidone palmitate; PP1M, paliperidone palmitate 1‐monthly; PP3M, paliperidone palmitate 3‐monthly; PSM, propensity score matching; SD, standard deviation; SMD, standardized mean difference.

^a^ One of the relapse event (psychiatric inpatient hospitalization, violent behavior, suicidal/homicidal ideation) did occur or present during baseline period (up to 1 year pre‐index PP injection period).

### 3.2 Relapse

Overall, out of 562 patients in each cohort, 114 (20.3%) in the PP3M cohort and 159 (28.3%) in the PP1M cohort had a relapse event, while 54 patients (9.6%) in the PP3M cohort and 78 patients (13.9%) in the PP1M cohort experienced relapse according to the censoring rules defined in the Japan label (Table 2). Inpatient hospitalizations due to psychiatric conditions were the most common reason for relapse (PP3M: 8.9%, PP1M: 9.8%; per Japan label censoring criteria: PP3M: 4.4%, PP1M: 4.8%; Table 2).

**TABLE 2:** Proportion of patients who had relapse and relapse criteria (PSM at 1:1 ratio).

Relapse definition	Proportion of patients who had relapse		Proportion of patients who had relapse per Japan label	
	PP3M	PP1M	PP3M	PP1M
Number accessed	562	562	562	562
Number of censored, *n* (%)	448 (79.7)	403 (71.7)	508 (90.4)	484 (86.1)
Number of relapse, *n* (%)	114 (20.3)	159 (28.3)	54 (9.6)	78 (13.9)
Relapse‐free status^a^, %	78.4	70.0	85.7	77.9
Difference (PP3M‐PP1M)	8.4		7.8	
95% CI^b^	(3.1–13.7)		(1.7–13.9)	
Reason of relapse, *n* (%)				
Violent behavior resulted in suicide	22 (3.9)	31 (5.5)	14 (2.5)	20 (3.6)
Suicidal ideation	5 (0.9)	12 (2.1)	3 (0.5)	8 (1.4)
Homicidal ideation	3 (0.5)	3 (0.5)	1 (0.2)	1 (0.2)
Suicidal and homicidal ideation	1 (0.2)	1 (0.2)	1 (0.2)	0 (0.0)
Deliberate self‐injection/violent behavior	3 (0.5)	1 (0.2)	0 (0.0)	1 (0.2)
Other^c^	1 (0.2)	2 (0.4)	0 (0.0)	0 (0.0)
Inpatient psychiatric hospitalization (IPH) alone	50 (8.9)	55 (9.8)	25 (4.4)	27 (4.8)
IPH + Homicidal ideation	4 (0.7)	10 (1.8)	0 (0.0)	5 (0.9)
IPH + Deliberate self‐injection/violent behavior	3 (0.5)	1 (0.2)	0 (0.0)	0 (0.0)
IPH + Violent behavior resulted in suicide	2 (0.4)	14 (2.5)	0 (0.0)	6 (1.1)
IPH + Suicidal and homicidal ideation	1 (0.2)	3 (0.5)	1 (0.2)	3 (0.5)
IPH + Suicidal ideation	18 (3.2)	26 (4.6)	9 (1.6)	7 (1.2)
IPH + Suicidal ideation/violent behavior	1 (0.2)	0 (0.0)	0 (0.0)	0 (0.0)

Abbreviations: CI, confidence interval; IPH, inpatient psychiatric hospitalization; PP1M, paliperidone palmitate 1‐monthly; PP3M, paliperidone palmitate 3‐monthly; PSM, propensity score matching.

^a^ Kaplan–Meier estimate.

^b^ Non‐inferiority of PP3M to PP1M will be concluded if the lower limit of the two‐sided 95% CI of the difference in proportion of patients who were relapse free between PP3M and PP1M exceeded the preselected non‐inferiority margin of −10%. PP3M will be declared superior to PP1M if the lower limit of the two‐sided 95% CI of the difference in the proportion of patients who were relapse free between PP3M and PP1M exceeded 0%.

^c^ Suicidal ideation, deliberate self‐injection/violent behavior, and violent behavior resulted in suicide.

The primary analysis for efficacy demonstrated that PP3M was non‐inferior to PP1M as measured by the percentage of subjects who remained relapse free at end of the 24‐month follow‐up period based on the Kaplan–Meier estimate. The estimated difference (95% CI) between the treatment groups (PP3M – PP1M) in percentages of subjects who remained relapse free was 8.4% (3.1–13.7) at month‐24. The lower bound of the 95% confidence interval (3.1%) was greater than the prespecified non‐inferiority margin of −10% demonstrating that PP3M was non‐inferior to PP1M. Similarly, for the Japan label consistent analysis, the differences in estimated relapse‐free rates were 7.8% (1.7–13.9), favoring PP3M (Table 2).

**FIGURE 3:**
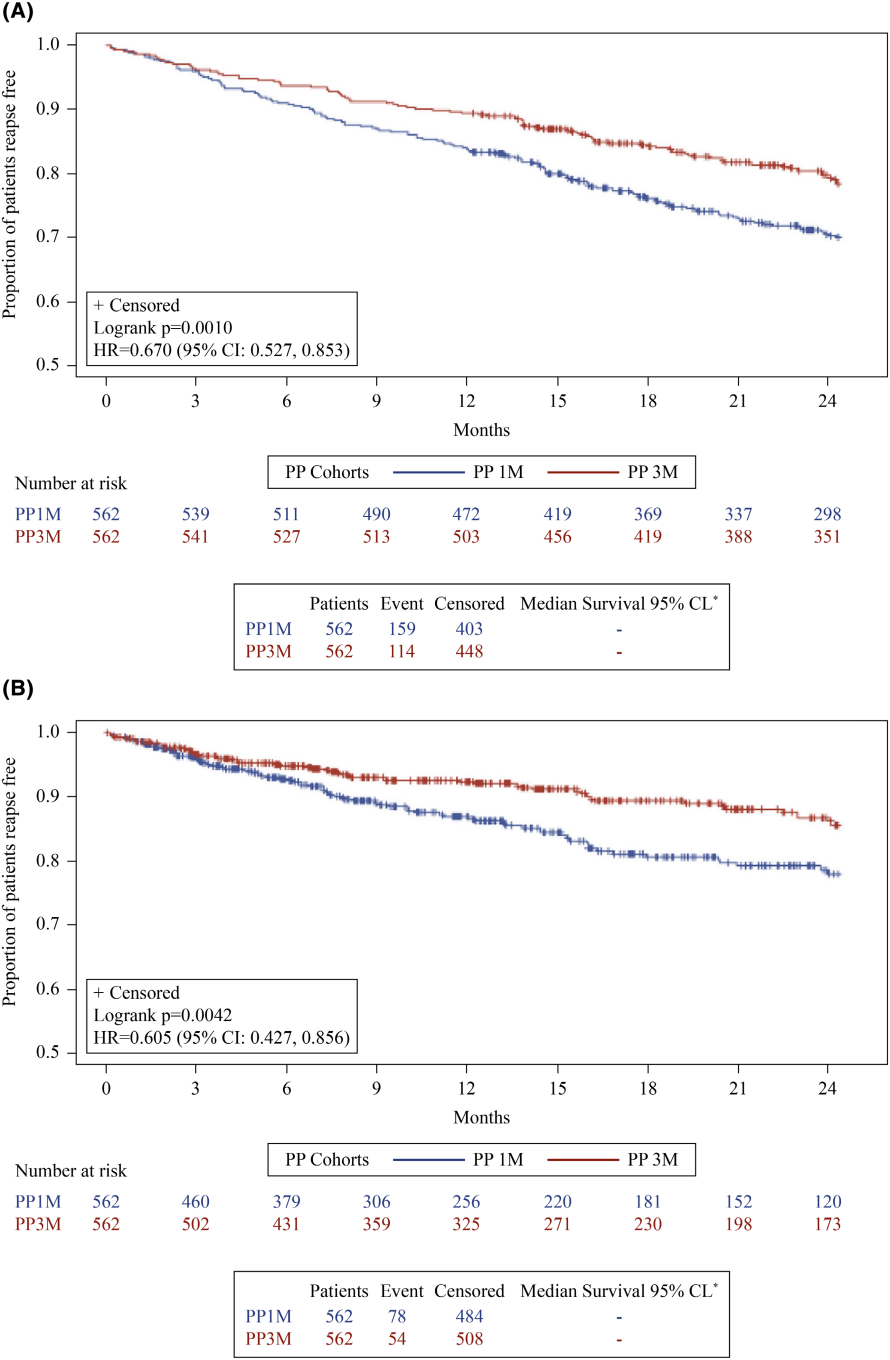
Proportion of relapse free patients and time to relapse (PSM 1:1). (A) Time to first relapse and proportion of relapse‐free patients. (B) Time to first relapse and proportion of relapse‐free patients per Japan label censoring. *Median time to relapses could not be calculated as 50% of patients did not have a relapse. CI, confidence interval; HR, hazard ratio; PP, paliperidone palmitate; PP1M, paliperidone palmitate 1‐monthly; PP3M, paliperidone palmitate 3‐monthly, PSM, propensity score matching.

Risk of relapse was significantly lower in the PP3M cohort compared with PP1M cohort (HR: 0.670; 95% CI: 0.527–0.853) as shown in Figure 3A. Similarly, risk of relapse was significantly lower in the PP3M cohort compared with PP1M cohort when Japan label censoring rules were applied (HR: 0.605; 95% CI: 0.427–0.856), Figure 3B.

### 3.3 Treatment persistence and adherence

**FIGURE 4:**
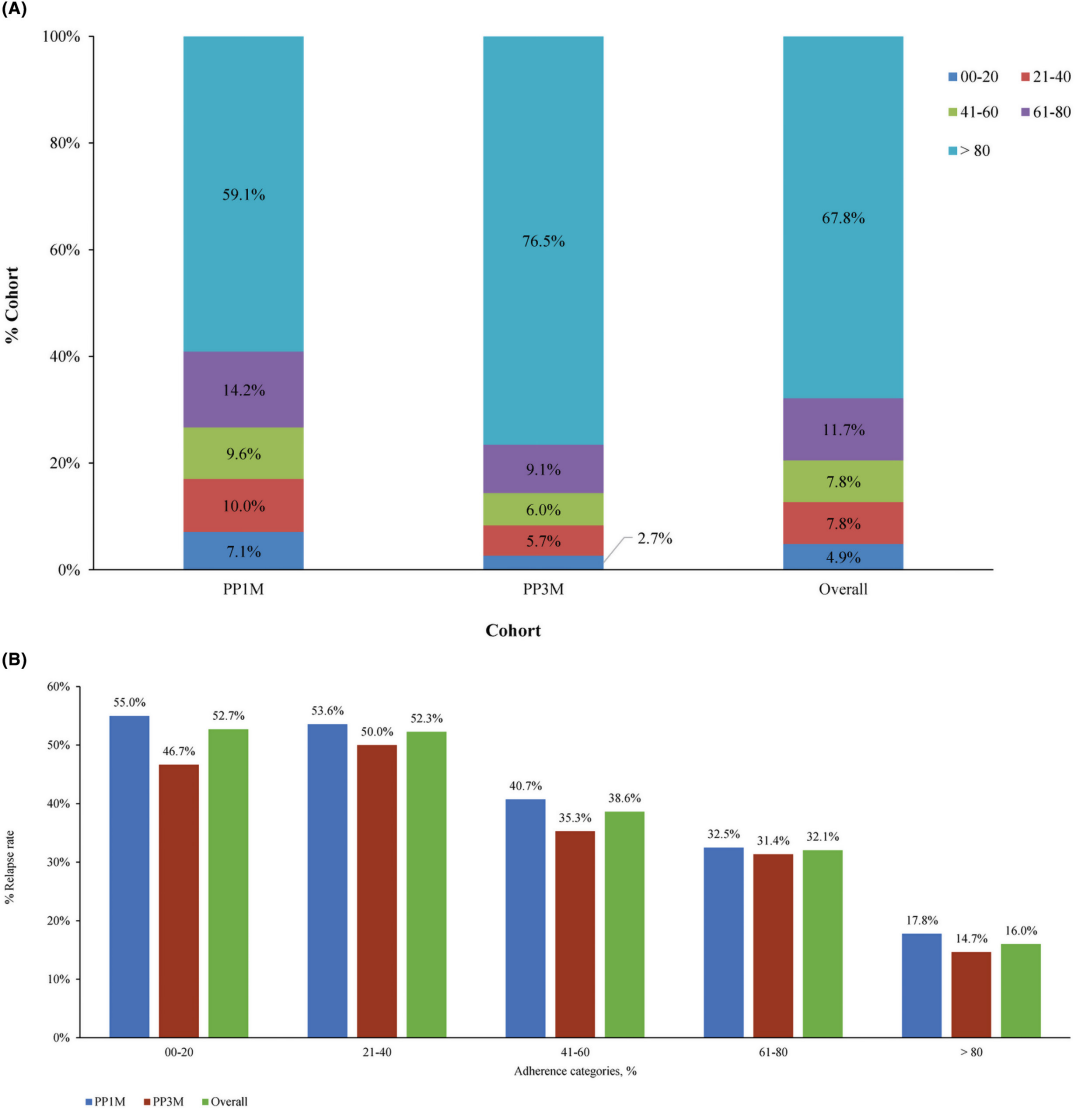
Treatment adherence (PSM 1:1). (A) Distribution of cohorts (%) by PP1M/PP3M adherence. (B) Distribution of relapse rates by PP1M/PP3M adherence categories. PP1M, paliperidone palmitate 1‐monthly; PP3M, paliperidone palmitate 3‐monthly, PSM, propensity score matching.

PP3M patients had a longer mean duration of treatment persistence than PP1M patients (Table 3) and this finding was consistent among all the three approaches used to calculate persistence. In addition, PP3M patients had a significantly higher PDC than PP1M patients. Patients were categorized into five groups based on the PDC on therapy (0%–20%, 21%–40%, 41%–60%, 61%–80%, and >80%). The percentage of patients in the first four PDC categories (0%–80%) were lower in PP3M cohort, while this percentage increased in the fifth category (>80% adherence) in the PP3M category (Figure 4A). This implies that the level of adherence was higher in the PP3M cohort (76.5%) compared to the PP1M cohort (59.1%) (Figure 4A). Relapse rate was highest among patients in the PDC category 0%–20% and lowest in >80%. Relapse rates were lower in the PP3M cohort compared to PP1M cohort in all the five PDC categories (Figure 4B).

**TABLE 3:** Treatment persistence (PSM at 1:1 ratio).

	PP3M (*n* = 562)	PP1M (*n* = 562)
Days with continuous supply, mean (SD)		
Injection with any PP formulation and dose strength	343.1 (238.1)	228.5 (213.6)
Injection with the same PP dose strength	328.5 (231.8)	212.5 (206.6)
Injection with the same PP formulation and dose strength	319.5 (231.7)	208.9 (204.5)

Abbreviations: PP, paliperidone palmitate; PP1M, paliperidone palmitate 1‐monthly; PP3M, paliperidone palmitate 3‐monthly; PSM, propensity score matching; SD, standard deviation.

### 3.4 Additional analyses

Additional analyses were performed to assess robustness of original findings using PSM at 1:2 ratio for PP3M and PP1M cohorts, respectively. Details of before and after PSM patient demographics and other baseline covariates used in PSM and their SMDs are presented in Supplementary Figure 1 and Supplementary Table 5. After PSM 1:2, 562 patients (female: 22.1%, mean age: 37.7 years) were included in the PP3M cohort and 1049 patients (female: 21.7%, mean age: 37.3 years) were included in the PP1M cohort (Supplementary Table 5). Most of the baseline characteristics were similar to the main analysis (Supplementary Table 5). Elixhauser and CCI domains with their SMDs are presented in Supplementary Table 6.

Overall, 114 patients (20.3%) in the PP3M cohort and 277 patients (26.4%) in the PP1M cohort had a relapse event, while 54 patients (9.6%) in the PP3M cohort and 133 patients (12.7%) in the PP1M cohort experienced relapse based on the Japan label (Supplementary Table 7). Risk of relapse was significantly lower in the PP3M cohort compared with PP1M cohort (HR: 0.730; 95% CI: 0.587–0.908) (Supplementary Figure 2A). Similarly, risk of relapse was significantly lower in the PP3M cohort compared with PP1M cohort (HR: 0.666; 95% CI: 0.485–0.914) (Supplementary Figure 2B) based on the Japan label.

PP3M patients had a significantly (*p* <0.0001) higher PDC than PP1M patients. Patients were categorized into five groups based on the PDC on therapy (0%–20%, 21%–40%, 41%–60%, 61%–80%, and >80%). The percentage of patients in the first four categories were lower in PP3M cohort, while the percentage increased in the fifth category (>80% adherence category) in the PP3M category (Supplementary Figure 3A). This implies that level of adherence was higher among the PP3M cohort (76.5%) compared to the PP1M cohort (60.9%) (Supplementary Figure 3A). Relapse rate was highest among patients in the PDC category 0%–20% and lowest in >80%. Relapse rates were comparatively lower in the PP3M cohort compared to PP1M cohort in the four PDC categories (0%–20%, 41%–60%, 61%–80%, and >80%) and were similar in both PP3M and PP1M cohorts in the 21%–40% PDC category (50%) (Supplementary Figure 3B).

## 4 DISCUSSION

This study used the MDCD database to evaluate the non‐inferiority of PP1M and PP3M monotherapy in maintaining relapse‐free status and to describe treatment persistence and adherence in patients with schizophrenia. Both primary (PSM at 1:1 ratio) and sensitivity (PSM at 1:2 ratio) analyses consistently revealed that a smaller proportion of patients experienced relapses in the PP3M group compared to PP1M. Moreover, the PP3M cohort exhibited a significantly decreased probability of relapse, with a notable 33% reduction compared to the PP1M cohort (HR: 0.67, 95% CI: 0.527–0.853). These findings remained robust even when considering the Japan label restrictions. The results not only confirmed the non‐inferiority of PP3M compared to PP1M therapy, but also demonstrated the superiority of PP3M over PP1M therapy in a RW setting. Furthermore, it was observed that PP3M showed higher treatment persistence and adherence compared to PP1M. This finding suggests that the superior relapse prevention effects of PP3M is possibly, at least in part, attributed to its great treatment persistence and adherence in the RW setting.

In the previous phase 3 randomized controlled trial (RCT), PP3M was reported to be non‐inferior versus PP1M (incidence of relapse: 8.1% [PP3M] vs. 9.2% [PP1M]).^25^ Our results not only validated the previous non‐inferiority findings, but also demonstrated the clinical superiority of PP3M over PP1M in a RW setting. Possible reasons for the difference between our study and the previous RCT could include variations in patient selection, differences in duration of stabilization periods, treatment adherence, and the differences in identification of relapse events in RCTs and RW setting including Positive and Negative Syndrome Scale total score‐based worsening as a relapse in RCTs. Moreover, it is important to note that the previous RCT was designed only as a non‐inferiority trial rather than a superiority trial. Various measures, such as placebo injections between two PP3M injections, were implemented to maintain unblinding.

Our study findings were consistent with the findings reported by the Li et al. study, which also conducted a retrospective health claims database analysis. Consistent with our study, Li et al^26^ employed a similar PS‐matching study to address baseline confounding factors when comparing PP3M versus PP1M. However, it is important to note that their study allowed for a more liberal use of concomitant antipsychotic medications, dosage adjustment in LAI, and off‐label LAI treatments. Incidence rate of relapse (13.81 per 100 person‐years in the PP1M cohort and 8.98 per 100 per‐years in the PP3M cohort) was similar to our results (relapse‐free rate at month 24: 78.4% in the PP3M cohort and 77.0% in the PP1M cohort). Notably, the comparative effectiveness of PP3M versus PP1M in relapse prevention in Li et al's main analysis (HR: 0.65) was quite similar to the results of our study, despite our analysis being conducted in a narrower patient population. These results confirm that while PP1M is effective in reducing the risk of relapse, treatment with PP3M, which has a longer dosing interval, appears to further reduce the risk of relapse.

In this study, hospitalization was the primary cause of relapse, which is in accordance with existing literature on relapse outcomes in schizophrenia.^37^ Multiple RW evidence studies using large‐scale RWD have shown that PP1M, along with other LAI treatments, is one of the most effective antipsychotic treatments for preventing psychiatric hospitalization in patients with schizophrenia.^38^, ^39^ Nevertheless, the clinical effectiveness of PP3M in preventing hospitalization was not specifically assessed in these earlier studies. Our study contributes to the growing body of evidence that demonstrates the effectiveness of PP3M in preventing hospitalization.^35^, ^40^, ^41^ Additionally, it is important to acknowledge that hospitalization does not capture all possible instances of relapse events in patients with schizophrenia.^42^ To address this limitation, our study employed a comprehensive definition of relapse, based on claims data, to capture as many relapse events as possible. However, it is to be noted that relapse events are captured by medical codes, and it has its own limitations.

To promote long‐term recovery in patients with schizophrenia, it is crucial to ensure proper treatment adherence to antipsychotic medications.^35^ Existing literature has shown that patients with schizophrenia and other psychiatric diseases exhibit improved treatment adherence when subject to less frequent dosing regimens.^43^ In our current analysis, we found that the PP3M cohort had a longer duration of treatment persistence and a higher proportion of patients with good adherence (PDC ≥80%) compared to the PP1M cohort. Additionally, a higher level of adherence to either PP1M or PP3M was associated with a reduced risk for relapse. Notably, even among patients within the same adherence category, the proportion of relapse was numerically lower in the PP3M cohort as compared to the PP1M cohort. The clinical significance of this finding is still uncertain. However, these results align with previous findings suggesting that the longer half‐life of PP3M may provide a more enduring effect in preventing relapse, even after its discontinuation.^44^ The longer half‐life of PP3M potentially offers benefits in mitigating the impact of nonadherence or early treatment discontinuation, which are common challenges encountered in patients receiving monthly LAI injections.^45^

A strength of this study is the use of PSM to create more balanced comparisons between PP3M and PP1M cohorts. This method helps to control the potential confounders and increases the internal validity of the study. However, due to its retrospective design and use of claims databases, this study has certain limitations. These include the possibility of incorrect diagnosis classification, data entry mistakes, a lack of clinical data (such as symptoms and disease severity), and a population that was skewed in terms of enrollment based on economic status. As there was no information available on patient's symptom severity measured using formal rating scales in the claims data, difference in symptom severity between PP1M and PP3M was not explored. Furthermore, the definition of relapse in our study was narrower compared to the relapse definition used in the previous PP3M clinical trials. Another potential limitation of this study is the generalizability of the results to other patient populations. The previous analysis of MDCD^26^ comparing the PP3M and PP1M groups included a wider range of patients due to fewer exclusion criteria, while this study focuses only on patients who were not receiving concomitant oral antipsychotic before and during PP1M/PP3M treatment. Although the analyzed patient population in this study aligns with the approved PP3M label in Japan, it is still important to consider whether the results can be adequately applied to the Japanese patient population, as the healthcare systems and patient population in Japan and the United States may differ in various aspects. Lastly, the study findings from Medicaid enrollees, who have low socioeconomic status, may not apply to those with higher socioeconomic status. In addition, RWD cannot control unmeasured or unknown confounders; they are restricted to known confounders that are available in both the trial data and the claims database. However, RWD also reflects RW patterns of care and vary in data quality and completeness.

## 5 CONCLUSION

In this analysis of RWD from the MDCD, patients with schizophrenia who switched to PP3M monotherapy (as aligned with the indication in the Japan label) had significantly lower risk of relapse compared to patients who continued treatment with PP1M monotherapy. Antipsychotic polypharmacy during LAI treatment remains a common practice worldwide including Japan.^46^ Concomitant antipsychotic use should be minimized or avoided during LAI treatment to ensure patient safety. The findings in this study offer valuable insights into the potential advantages of using PP3M as monotherapy compared to treatment with PP1M, aligning with the specified conditions in Japan's label. Since the MDCD claims database included patients outside Japan, further studies are warranted to establish the reduction in relapse rate among Japanese patients with schizophrenia in RW setting.

## AUTHOR CONTRIBUTIONS

Chih‐Lin Chiang, Madoka Chinen, Akihide Wakamatsu, and Ibrahim Turkoz were involved in conception and design of the study. Mehmet Daskiran and Ibrahim Turkoz were involved in the acquisition and analysis of data. All authors contributed to the data interpretation for the results. All authors drafted the manuscript. All authors had access to the study data, provided direction and comments on the manuscript draft, made the final decision about where to publish these data, and approved submission to the journal.

## FUNDING INFORMATION

This study was sponsored by Janssen Pharmaceutical K.K.

## CONFLICT OF INTEREST STATEMENT

All the authors are employees of Janssen and its subsidiaries during the study and may own stock or stock options. The authors report no other conflicts of interest in this work.

## ETHICS STATEMENT

Approval of the Research Protocol by an Institutional Reviewer Board: N/A.

Informed Consent: N/A.

Registry and the Registration No. of the Study/Trial: N/A.

Animal Studies: N/A.

## Supporting information


Data S1.

## Data Availability

The MDCD data used in this study are available from Merative at https://www.merative.com/documents/brief/marketscan‐explainer‐general.
