# Supplementary material for: Clinical effectiveness of paliperidone palmitate 3‐monthly and 1‐monthly as monotherapy in patients with schizophrenia: A retrospective cohort study based on the Medicaid claims database
Source: Neuropsychopharmacol Rep. 2024 Sep 11;44(4):716–27. doi: 10.1002/npr2.12473 (PMC11609742; doi:10.1002/npr2.12473)
Supplement: Supplementary file 1 — Data S1. [file NPR2-44-716-s001.docx]

**Supplementary Information**

**Supplementary Table 1.** Codes for identifying diagnoses, medication records and procedures

|  | **ICD 10** | **ICD 9** | **Code description** |
| --- | --- | --- | --- |
| Suicidal ideation | R45.851 | V62.84 |  |
| Suicide Attempt (Self-Harm) | X60 -X84 | E950 E951 E952 E953 E954 E955 E956 E957 E958 E959 | Intentional self-harm (X60-X84). Includes: purposely self-inflicted poisoning or injury suicide (attempted) |
|  | Y87.0 |  | Sequelae of intentional self-harm |
|  | T14.91 |  | Suicide attempt |
| Injury, undetermined intents |  | E980 E981 E982 E983 E984 |  |
|  | Y21 |  | Drowning and submersion, undetermined intent |
|  | Y22-Y28, Y30-Y33 | E985 E986 E987 E988 E989 | Event of undetermined intent (Y10-Y34) |
|  | Y29 |  | Contact with blunt object, undetermined intent |
|  | Y34 |  | ‘Unspecified event, undetermined intent’ |
|  | Y10-Y15 |  | ‘undetermined intent’ |
|  | Y16 |  | Poisoning by and exposure to organic solvents and halogenated hydrocarbons and their vapors, undetermined intent |
|  | Y17 |  | Poisoning by and exposure to other gases and vapors, undetermined intent |
|  | Y18 |  | Poisoning by and exposure to pesticides, undetermined intent |
|  | Y19 |  | Poisoning by and exposure to other and unspecified chemicals and noxious substances, undetermined intent |
|  | Y20 |  | Hanging, strangulation and suffocation, undetermined intent |
| Violent behavior | R45.6 | 300.9 | Violent behavior |
| Hostility | R45.5 | 300.9 | Tendency to feel anger toward and to seek to inflict harm upon a person or group |
| Homicidal ideation | R45.850 | V62.85 | Homicidal ideation, homicidal thoughts, ideation homicidal |
| Aggressive behavior | F03.91 | 312 | Aggressive unsocial conduct disorder, angry, behavior disorder, aggressive type unsocialized, feeling angry, unsocialized behavior, aggressive type |
| Aggressive behavior | F91.8 | 312.89, 312.10 | F91.8 is grouped within Diagnostic Related Group (MS-DRG v34.0) |
| Schizophrenia diagnosis | | | |
|  |  | 295.7 | Schizoaffective disorder |
|  | F20.x |  | Schizophrenia |
|  |  | 295.70-295.74 | Schizoaffective disorder (unspecified, subchronic, chronic, subchronic with acute exacerbation, chronic with exacerbation) |
|  |  | 295.75 | Schizoaffective disorder, in remission |
|  | F25.8 | 295.8 | Other specified types of schizophrenia |
|  |  | 295.80-295.85 | Other specified types of schizophrenia (unspecified, subchronic, chronic, subchronic with acute exacerbation, chronic with acute exacerbation, in remission) |
|  | F25.9 | 295.9 | Unspecified schizophrenia |
|  |  | 295.90-295.95 | Unspecified schizophrenia (unspecified, subchronic, chronic, subchronic with acute exacerbation, chronic with acute exacerbation, in remission) |
|  | F25.0 |  | Schizoaffective disorder, bipolar type |
|  | F25.1 |  | Schizoaffective disorder, depressive type |
|  |  | 297 | Paranoid states (Delusional disorders) |
|  |  | 297.0 | Paranoid state, simple |
|  |  | 297.1 | Delusional disorder |
|  |  | 297.2 | Paraphrenia |
|  |  | 297.3 | Shared psychotic disorder |
|  |  | 297.8 | Other specified paranoid states |
| Mania and bipolar |  |  |  |
|  |  | 296.0 | Bipolar I disorder, single manic episode |
|  |  | 296.00-296.06 | Bipolar I disorder, single manic episode (unspecified, mild, moderate, severe without mention of psychotic behavior, severe specified as with psychotic behavior, in partial or unspecified remission, in remission) |
|  |  | 296.1 | Manic disorder, recurrent episode |
|  |  | 296.10-296.16 | Manic affective disorder, recurrent episode (unspecified, mild, moderate, severe without mention of psychotic behavior, severe specified as with psychotic behavior, in partial or unspecified remission, in remission) |
|  |  | 296.4 | Bipolar I disorder, most recent episode (or current) manic |
|  |  | 296.40-296.46 | Bipolar I disorder, most recent episode/current manic (unspecified, mild, moderate, severe without mention of psychotic behavior, severe specified as with psychotic behavior, in partial or unspecified remission, in remission) |
|  |  | 296.5 | Bipolar I disorder, most recent episode (or current) depressed |
|  |  | 296.50 -296.56 | Bipolar I disorder, most recent episode/current depressed (unspecified, mild, moderate, severe without mention of psychotic behavior, severe specified as with psychotic behavior, in partial or unspecified remission, in remission) |
|  |  | 296.6 | Bipolar I disorder, most recent episode (or current) mixed |
|  |  | 296.7 | Bipolar I disorder, most recent episode (or current) unspecified |
|  |  | 296.8 | Other and unspecified bipolar disorders |
|  | F30 |  | Mania |
|  | F31 |  | Bipolar disorder |
| Dementia |  |  |  |
|  |  | 46 | Kuru |
|  |  | 46.1 | Jakob-Creutzfeldt disease |
|  |  | 46.11 | Variant Creutzfeldt-Jakob disease |
|  |  | 46.19 | Other and unspecified Creutzfeldt-Jakob disease |
|  |  | 94.1 | General paresis |
|  |  | 290 | Dementias |
|  |  | 290.0 | Senile dementia, uncomplicated |
|  |  | 290.1 | Presenile dementia |
|  |  | 290.2 | Senile dementia with delusional or depressive features |
|  |  | 290.3 | Senile dementia with delirium |
|  |  | 290.4 | Vascular dementia |
|  |  | 290.8 | Other specified senile psychotic conditions |
|  |  | 290.9 | Unspecified senile psychotic condition |
|  |  | 291.1 | Alcohol-induced persisting amnestic disorder |
|  |  | 291.2 | Alcohol-induced persisting dementia |
|  |  | 292.82 | Drug-induced persisting dementia |
|  |  | 294 | Amnestic disorder in conditions classified elsewhere |
|  |  | 294.1 | Dementia in conditions classified elsewhere |
|  |  | 294.2 | Dementia, unspecified |
|  |  | 294.8 | Other persistent mental disorders due to conditions classified elsewhere |
|  |  | 294.9 | Unspecified persistent mental disorders due to conditions classified elsewhere |
|  |  | 310 | Frontal lobe syndrome |
|  |  | 310.2 | Post-concussion syndrome |
|  |  | 330 | Cerebral degenerations usually manifest in childhood |
|  |  | 330.1 | Cerebral lipidoses |
|  |  | 330.2 | Cerebral degeneration in generalized lipidoses |
|  |  | 330.3 | Cerebral degeneration of childhood, in other diseases classified elsewhere |
|  |  | 330.8 | Other specified cerebral degenerations in childhood |
|  |  | 330.9 | Unspecified cerebral degenerations in childhood |
|  |  | 331 | Alzheimer's disease |
|  |  | 331.1 | Frontotemporal dementia |
|  |  | 331.11 | Pick's disease |
|  |  | 331.19 | Other frontotemporal dementia |
|  |  | 331.2 | Senile degeneration of brain |
|  |  | 331.3 | Communicating hydrocephalus |
|  |  | 331.4 | Obstructive hydrocephalus |
|  |  | 331.5 | Idiopathic normal pressure hydrocephalus |
|  |  | 331.7 | Cerebral degeneration in diseases classified elsewhere |
|  |  | 331.8 | Other cerebral degeneration |
|  |  | 333.4 | Huntington's chorea |
|  |  | 780.93 | Memory loss |
|  |  | 797 | Senility without mention of psychosis |
| Autism | Z91.83, F84.0 | 299.0 |  |
| Depressive disorder | | | |
|  |  | 296.2, 296.20, 296.21, 296.22, 296.23, 296.24 | Major depressive disorder, single episode |
|  |  | 296.3, 296.30, 296.31, 296.32, 296.33, 296.34 | Major depressive disorder, recurrent episode |
|  |  | 300.4, 300.40, 309.0, 309.00, 309.1, 309.10 | Dysthymic disorder, dysthymia specify prim |
|  |  | 311, 311.0, 311.00 | Depressive disorder, NOS |
|  | F32, F33 or F34 |  | depression: |
| Substance abuse |  |  |  |
|  |  | 291 | Alcohol-induced mental disorders |
|  |  | 291.0 | Alcohol withdrawal delirium |
|  |  | 291.3 | Alcohol-induced psychotic disorder with hallucinations |
|  |  | 291.4 | Idiosyncratic alcohol intoxication |
|  |  | 291.5 | Alcohol-induced psychotic disorder with delusions |
|  |  | 291.8 | Other specified alcohol-induced mental disorders |
|  |  | 291.81 | Alcohol withdrawal |
|  |  | 291.82 | Alcohol induced sleep disorders |
|  |  | 291.89 | Other alcohol-induced mental disorders |
|  |  | 291.9 | Unspecified alcohol-induced mental disorders |
|  |  | 292 | Drug-induced mental disorders |
|  |  | 292.1 | Drug-induced psychotic disorders |
|  |  | 292.11 | Drug-induced psychotic disorder with delusions |
|  |  | 292.12 | Drug-induced psychotic disorder with hallucinations |
|  |  | 292.8 | Other specified drug-induced mental disorders |
|  |  | 292.9 | Unspecified drug-induced mental disorder |
|  |  | 293 | Delirium due to conditions classified elsewhere |
|  |  | 293.1 | Subacute delirium |
|  |  | 293.81 | Psychotic disorder with delusions in conditions classified elsewhere |
|  |  | 296 | Episodic mood disorders |
|  |  | 296.9 | Other and unspecified episodic mood disorder |
|  |  | 296.90 | Unspecified episodic mood disorder |
|  |  | 298.2 | Reactive confusion |
|  |  | 301.1 | Affective personality disorder |
|  |  | 301.13 | Cyclothymic disorder |
|  |  | 303 | Alcohol dependence syndrome |
|  |  | 303.9 | Other and unspecified alcohol dependence |
|  |  | 305.8 | Antidepressant type abuse |
|  |  | 437.7 | Transient global amnesia |
|  | V11.3 |  | Personal history of alcoholism |

**Supplementary Table 2:** Equivalent PP1M and PP3M dose

| **Strength level** | **PP1M dose** | **PP3M dose** |
| --- | --- | --- |
| 1 | 50 mg eq. (NDC 50458-562-01) | 175 mg eq. (NDC 50458-606-01) |
| 2 | 75 mg eq. (NDC 50458-562-01) | 263 mg eq. (NDC 50458-607-01) |
| 3 | 100 mg eq. (NDC 50458-563-01) | 350 mg eq. (NDC 50458-608-01) |
| 4 | 150 mg eq. (NDC 50458-564-01) | 525 mg eq. (NDC 50458-609-01) |
| NDC, National Drug Code; PP1M, paliperidone palmitate once-monthly; PP3M, paliperidone palmitate 3-monthly | | |

**Supplementary Table 3.** Patient attrition summary

| **Attrition** | **All patients** | **PP3M** | **PP1M** |
| --- | --- | --- | --- |
| Merative™ MarketScan^®^ Multi-State Medicaid population (2000-2022) | 35971295 | - | **-** |
| Merative™ MarketScan^®^ Multi-State Medicaid population (2015-2022) | 25052380 | **-** | **-** |
| Inclusion 1. Patients with ≥1 LAI PP record | 43693 | 8040 | 42218 |
| Inclusion 2. Patients with ≥1 schizophrenia diagnosis at or before their first PP3M injection | 29890 | 5302 | 28626 |
| Inclusion 3. Patients must be ≥18 at first PP3M Injection | 29521 | 5292 | 28471 |
| Exclusion 1. Exclude patients with autism, dementia or Bipolar diagnosis prior to index PP injection | 20392 | 4110 | 19306 |
| Exclusion 2. Exclude patients with clozapine or treatment affecting PP metabolism during baseline | 19511 | 3943 | 18445 |
| Exclusion 3. Exclude patients with cancer, pregnant, or morbidly obese / underweight diagnosis during baseline | 18158 | 3662 | 17115 |
| Inclusion 4. Include patients with at least 1 year pre- and post-index insurance enrolment | 9868 | 2022 | 9343 |
| Inclusion 5. Include adequately treated PP3M patients only and apply the same for PP1M cohort | 4644 | 618 | 4564 |
| Exclusion 4. Exclude patients with oral and/or other injectable LAI during last 6 month of baseline | 4252 | 582 | 4167 |
| Exclusion 5. Exclude patients from PP1M cohort if they are eligible for both cohorts | 4252 | 582 | 3670 |

LAI, long-acting injectable; PP, paliperidone palmitate; PP1M, Paliperidone palmitate 1-monthly; PP3M, Paliperidone palmitate 3-monthly

**Supplementary Table 4.** Details of CCI and Elixhauser comorbidity index domains before and after PSM (1:1) and SDM

| **Number of patients, n (%)** | **Before Matching** | | | **After Matching** | | |
| --- | --- | --- | --- | --- | --- | --- |
|  | **PP3M** | **PP1M** | **SMD** | **PP3M** | **PP1M** | **SMD** |
|  | **n=582** | **n=3,670** |  | **n=562** | **n=562** |  |
| **CCI domains** |  |  |  |  |  |  |
| Myocardial Infarction | 5 (0.8) | 35 (0.9) | 0.010 | 5 (0.8) | 3 (0.5) | 0.042 |
| Congestive Heart Failure | 5 (0.8) | 65 (1.7) | 0.080 | 5 (0.8) | 3 (0.5) | 0.042 |
| Peripheral Vascular Disease | 16 (2.7) | 65 (1.7) | 0.066 | 14 (2.4) | 5 (0.8) | 0.124 |
| Cerebrovascular Disease | 8 (1.3) | 46 (1.2) | 0.011 | 7 (1.2) | 6 (1.0) | 0.017 |
| Dementia | 0 (0.0) | 0(0.0) | 0.000 | 0 (0.0) | 0 (0.0) | 0.000 |
| Chronic Pulmonary Disease | 84 (14.4) | 571 (15.5) | 0.032 | 79 (14.0) | 62 (11.0) | 0.091 |
| Connective Tissue Disease-Rheumatic Disease | 0 (0.0) | 23 (0.6) | 0.112 | 0 (0.0) | 0 (0.0) | 0.000 |
| Peptic Ulcer Disease | 2 (0.3) | 18 (0.4) | 0.023 | 2 (0.3) | 3 (0.5) | 0.027 |
| Mild Liver Disease | 15 (2.5) | 132 (3.5) | 0.059 | 14 (2.4) | 16 (2.8) | 0.022 |
| Diabetes without complications | 58 (9.9) | 473 (12.8) | 0.092 | 52 (9.2) | 53 (9.4) | 0.006 |
| Diabetes with complications | 15 (2.5) | 116 (3.1) | 0.035 | 12 (2.1) | 8 (1.4) | 0.054 |
| Paraplegia and Hemiplegia | 0 (0.0) | 9 (0.2) | 0.070 | 0 (0.0) | 1 (0.1) | 0.060 |
| Renal Disease | 8 (1.3) | 69 (1.8) | 0.040 | 8 (1.4) | 5 (0.8) | 0.050 |
| Cancer | 0 (0.0) | 0 (0.0) | 0.000 | 0 (0.0) | 0 (0.0) | 0.000 |
| Moderate or Severe Liver Disease | 0 (0.0) | 6 (0.1) | 0.057 | 0 (0.0) | 0 (0.0) | 0.000 |
| Metastatic Carcinoma | 0 (0.0) | 0 (0.0) | 0.000 | 0 (0.0) | 0 (0.0) | 0.000 |
| AIDS/HIV | 4 (0.6) | 36 (0.9) | 0.032 | 4 (0.7) | 5 (0.8) | 0.020 |
| **Elixhauser comorbidity index domains** |  |  |  |  |  |  |
| Congestive Heart Failure | 5 (0.8) | 65 (1.7) | 0.080 | 5 (0.8) | 3 (0.5) | 0.042 |
| Cardiac Arrhythmia | 39 (6.7) | 385 (10.4) | 0.136 | 37 (6.5) | 34 (6.0) | 0.022 |
| Valvular Disease | 9 (1.5) | 45 (1.2) | 0.027 | 9 (1.6) | 1 (0.1) | 0.152 |
| Pulmonary Circulation Disorders | 3 (0.5) | 28 (0.7) | 0.031 | 3 (0.5) | 0 (0.0) | 0.104 |
| Peripheral Vascular Disorders | 16 (2.7) | 65 (1.7) | 0.066 | 14 (2.4) | 5 (0.8) | 0.124 |
| Hypertension Uncomplicated | 120 (20.6) | 956 (26.0) | 0.129 | 116 (20.6) | 109 (19.3) | 0.031 |
| Hypertension Complicated | 14 (2.4) | 73 (1.9) | 0.028 | 14 (2.4) | 4 (0.7) | 0.142 |
| Paralysis | 0 (0.0) | 9 (0.2) | 0.070 | 0 (0.0) | 1 (0.1) | 0.060 |
| Other Neurological Disorders | 21 (3.6) | 202 (5.5) | 0.091 | 19 (3.3) | 18 (3.2) | 0.010 |
| Chronic Pulmonary Disease | 84 (14.4) | 571 (15.5) | 0.032 | 79 (14.0) | 62 (11.0) | 0.091 |
| Diabetes Uncomplicated | 55 (9.4) | 446 (12.1) | 0.087 | 50 (8.8) | 51 (9.0) | 0.006 |
| Diabetes Complicated | 26 (4.4) | 237 (6.4) | 0.088 | 21 (3.7) | 23 (4.0) | 0.018 |
| Hypothyroidism | 18 (3.0) | 191 (5.2) | 0.106 | 17 (3.0) | 23 (4.0) | 0.058 |
| Renal Failure | 8 (1.3) | 69 (1.8) | 0.040 | 8 (1.4) | 5 (0.8) | 0.050 |
| Liver Disease | 15 (2.5) | 136 (3.7) | 0.065 | 14 (2.4) | 16 (2.8) | 0.022 |
| Peptic Ulcer Disease excluding bleeding | 2 (0.3) | 18 (0.4) | 0.023 | 2 (0.3) | 3 (0.5) | 0.027 |
| AIDS/HIV | 4 (0.6) | 36 (0.9) | 0.032 | 4 (0.7) | 5 (0.8) | 0.020 |
| Lymphoma | 0 (0.0) | 0 (0.0) | 0.000 | 0 (0.0) | 0 (0.0) | 0.000 |
| Metastatic Cancer | 0 (0.0) | 0 (0.0) | 0.000 | 0 (0.0) | 0 (0.0) | 0.000 |
| Solid Tumor without Metastasis | 0 (0.0) | 0 (0.0) | 0.000 | 0 (0.0) | 0 (0.0) | 0.000 |
| Rheumatoid Arthritis/collagen | 1 (0.1) | 37 (1.0) | 0.109 | 1 (0.1) | 0 (0.0) | 0.060 |
| Coagulopathy | 9 (1.5) | 40 (1.0) | 0.040 | 9 (1.6) | 4 (0.7) | 0.083 |
| Obesity | 60 (10.3) | 423 (11.5) | 0.039 | 57 (10.1) | 53 (9.4) | 0.024 |
| Weight Loss | 8 (1.3) | 28 (0.7) | 0.060 | 7 (1.2) | 1 (0.1) | 0.127 |
| Fluid and Electrolyte Disorders | 31 (5.3) | 302 (8.2) | 0.116 | 29 (5.1) | 27 (4.8) | 0.016 |
| Blood Loss Anemia | 1 (0.1) | 15 (0.4) | 0.044 | 1 (0.1) | 3 (0.5) | 0.060 |
| Deficiency Anemia | 17 (2.9) | 122 (3.3) | 0.023 | 14 (2.4) | 16 (2.8) | 0.022 |
| Alcohol Abuse | 74 (12.7) | 568 (15.4) | 0.079 | 71 (12.6) | 74 (13.1) | 0.016 |
| Drug Abuse | 151 (25.9) | 1,201 (32.7) | 0.149 | 147 (26.1) | 175 (31.1) | 0.110 |
| Psychoses | 582 (100.0) | 3,670 (100.0) | 0.000 | 562 (100.0) | 562 (100.0) | 0.000 |
| Depression | 108 (18.5) | 987 (26.8) | 0.200 | 101 (17.9) | 100 (17.7) | 0.005 |

CCI, Charlson Comorbidity Index; PP1M, paliperidone palmitate 1-monthly; PP3M, paliperidone palmitate 3-monthly; PS, propensity score; SMD, standardized mean difference; SD, standard deviation

**Supplementary Table 5: Additional analysis-** Baseline demographics and disease state characteristics for PP3M and PP1M cohorts before and after PSM (PSM ratio 1:2)

| **Patient Characteristics** | **Before Matching** | | | **After Matching** | | |
| --- | --- | --- | --- | --- | --- | --- |
|  | **PP3M** | **PP1M** | **SMD** | **PP3M** | **PP1M** | **SMD** |
|  | **n=582** | **n=3,670** |  | **n=562** | **n=1049** |  |
| Age, mean (SD), years | 37.8 (12.14) | 38.6 (12.83) | 0.067 | 37.7 (12.07) | 37.3 (11.99) | 0.033 |
| Age category, n (%) |  |  |  |  |  |  |
| 18-25 years | 96 (16.5) | 583 (15.9) | 0.017 | 90 (16.0) | 162 (15.4) | 0.016 |
| 26-50 years | 378 (64.9) | 2224 (60.6) | 0.090 | 370 (65.8) | 702 (66.9) | 0.023 |
| ≥51 years | 108 (18.6) | 863 (23.5) | 0.122 | 102 (18.1) | 185 (17.6) | 0.013 |
| Sex, n (%) |  |  |  |  |  |  |
| Female | 132 (22.7) | 990 (27.0) | 0.100 | 124 (22.1) | 228 (21.7) | 0.008 |
| Race/ethnicity, n (%) |  |  |  |  |  |  |
| Black | 320 (55.0) | 1883 (51.3) | 0.074 | 310 (55.2) | 588 (56.1) | 0.018 |
| White | 131 (22.5) | 867 (23.6) | 0.026 | 130 (23.1) | 247 (23.5) | 0.010 |
| Mixed/unknown | 74 (12.7) | 468 (12.8) | 0.001 | 71 (12.6) | 125 (11.9) | 0.022 |
| Other | 37 (6.4) | 312 (8.5) | 0.082 | 31 (5.5) | 62 (5.9) | 0.017 |
| Hispanic | 9 (1.5) | 49 (1.3) | 0.018 | 9 (1.6) | 7 (0.7) | 0.088 |
| Missing | 11 (1.9) | 91 (2.5) | 0.04 | 11 (2.0) | 20 (1.9) | 0.004 |
| CCI score, mean (SD) | 0.5 (1.11) | 0.6 (1.17) | 0.094 | 0.4 (1.10) | 0.4 (0.92) | 0.069 |
| Elixhauser Comorbidity Index score, mean (SD) | 2.5 (1.79) | 3.0 (1.99) | 0.228 | 2.5 (1.77) | 2.4 (1.59) | 0.054 |
| Depressive disorder diagnosis | 105 (18.0) | 960 (26.2) | 0.197 | 99 (17.6) | 162 (15.4) | 0.059 |
| Index PP dose, n (%) |  |  |  |  |  |  |
| 175/50 mg eq. | 8 (1.4) | 54 (1.5) | 0.008 | 3 (0.5) | 3 (0.3) | 0.039 |
| 263/75 mg eq. | 99 (17.0) | 522 (14.2) | 0.077 | 94 (16.7) | 157 (15.0) | 0.048 |
| 350/100 mg eq. | 228 (39.2) | 1545 (42.1) | 0.06 | 224 (39.9) | 430 (41.0) | 0.023 |
| 525/150 mg eq. | 247 (42.4) | 1549 (42.2) | 0.005 | 241 (42.9) | 459 (43.8) | 0.018 |
| Prior relapse event^*^, n (%) | 97 (16.7) | 1105 (30.1) | 0.322 | 91 (16.2) | 164 (15.6) | 0.015 |
| Prior psychiatric inpatient hospitalization, n (%) | 82 (14.1) | 961 (26.2) | 0.305 | 76 (13.5) | 143 (13.6) | 0.003 |
| Prior mental health ER visit, n (%) | 67 (11.5) | 723 (19.7) | 0.227 | 65 (11.6) | 167 (15.9) | 0.137 |

^*^One of the relapse event (psychiatric inpatient hospitalization, violent behavior, suicidal / homicidal ideation) did occur or present during baseline period (up to 1 year pre-index PP injection period).

CCI, Charlson Comorbidity Index; eq., equivalent; ER, emergency room; PP, paliperidone palmitate; PP1M, paliperidone palmitate 1-monthly; PP3M, paliperidone palmitate 3-monthly; PSM, propensity score matching; SMD, standardized mean difference; SD, standard deviation.

**Supplementary Table 6. Additional analysis-** Details of CCI and Elixhauser comorbidity index domains before and after PSM (1:2) and SMD

| **Number of patients, n (%)** | **Before Matching** | | | **After Matching** | | |
| --- | --- | --- | --- | --- | --- | --- |
|  | **PP3M** | **PP1M** | **SMD** | **PP3M** | **PP1M** | **SMD** |
|  | **n=582** | **n=3,670** |  | **n=562** | **n=1049** |  |
| **CCI domains** |  |  |  |  |  |  |
| Myocardial Infarction | 5 (0.8) | 35 (0.9) | 0.010 | 5 (0.8) | 6 (0.5) | 0.037 |
| Congestive Heart Failure | 5 (0.8) | 65 (1.7) | 0.080 | 5 (0.8) | 6 (0.5) | 0.037 |
| Peripheral Vascular Disease | 16 (2.7) | 65(1.7) | 0.066 | 14 (2.4) | 8 (0.7) | 0.137 |
| Cerebrovascular Disease | 8 (1.3) | 46(1.2) | 0.011 | 7 (1.2) | 6 (0.5) | 0.071 |
| Dementia | 0 (0.0) | 0(0.0) | 0.000 | 0 (0.0) | 0 (0.0) | 0.000 |
| Chronic Pulmonary Disease | 84 (14.4) | 571(15.5) | 0.032 | 79 (14.0) | 104 (9.9) | 0.128 |
| Connective Tissue Disease-Rheumatic Disease | 0 (0.0) | 23 (0.6) | 0.112 | 0 (0.0) | 1 (0.0) | 0.044 |
| Peptic Ulcer Disease | 2 (0.3) | 18 (0.4) | 0.023 | 2 (0.3) | 5 (0.4) | 0.019 |
| Mild Liver Disease | 15 (2.5) | 132 (3.5) | 0.059 | 14 (2.4) | 28 (2.6) | 0.011 |
| Diabetes without complications | 58 (9.9) | 473 (12.8) | 0.092 | 52 (9.2) | 104 (9.9) | 0.022 |
| Diabetes with complications | 15 (2.5) | 116 (3.1) | 0.035 | 12 (2.1) | 18 (1.7) | 0.031 |
| Paraplegia and Hemiplegia | 0 (0.0) | 9 (0.2) | 0.070 | 0 (0.0) | 1 (0.0) | 0.044 |
| Renal Disease | 8 (1.3) | 69 (1.8) | 0.040 | 8 (1.4) | 9 (0.8) | 0.053 |
| Cancer | 0 (0.0) | 0 (0.0) | 0.000 | 0 (0.0) | 0 (0.0) | 0.000 |
| Moderate or Severe Liver Disease | 0 (0.0) | 6 (0.1) | 0.057 | 0 (0.0) | 0 (0.0) | 0.000 |
| Metastatic Carcinoma | 0 (0.0) | 0 (0.0) | 0.000 | 0 (0.0) | 0 (0.0) | 0.000 |
| AIDS/HIV | 4 (0.6) | 36 (0.9) | 0.032 | 4 (0.7) | 9 (0.8) | 0.017 |
| **Elixhauser comorbidity index domains** |  |  |  |  |  |  |
| Congestive Heart Failure | 5 (0.8) | 65 (1.7) | 0.080 | 5 (0.8) | 6 (0.5) | 0.037 |
| Cardiac Arrhythmia | 39 (6.7) | 385 (10.4) | 0.136 | 37 (6.5) | 77 (7.3) | 0.030 |
| Valvular Disease | 9 (1.5) | 45 (1.2) | 0.027 | 9 (1.6) | 3 (0.2) | 0.136 |
| Pulmonary Circulation Disorders | 3 (0.5) | 28 (0.7) | 0.031 | 3 (0.5) | 0 (0.0) | 0.104 |
| Peripheral Vascular Disorders | 16 (2.7) | 65 (1.7) | 0.066 | 14 (2.4) | 8 (0.7) | 0.137 |
| Hypertension Uncomplicated | 120 (20.6) | 956 (26.0) | 0.129 | 116 (20.6) | 216 (20.5) | 0.001 |
| Hypertension Complicated | 14 (2.4) | 73 (1.9) | 0.028 | 14 (2.4) | 9 (0.8) | 0.128 |
| Paralysis | 0 (0.0) | 9 (0.2) | 0.070 | 0 (0.0) | 1 (0.0) | 0.044 |
| Other Neurological Disorders | 21 (3.6) | 202 (5.5) | 0.091 | 19 (3.3) | 49 (4.6) | 0.066 |
| Chronic Pulmonary Disease | 84 (14.4) | 571 (15.5) | 0.032 | 79 (14.0) | 104 (9.9) | 0.128 |
| Diabetes Uncomplicated | 55 (9.4) | 446 (12.1) | 0.087 | 50 (8.8) | 98 (9.3) | 0.015 |
| Diabetes Complicated | 26 (4.4) | 237 (6.4) | 0.088 | 21 (3.7) | 44 (4.1) | 0.023 |
| Hypothyroidism | 18 (3.0) | 191 (5.2) | 0.106 | 17 (3.0) | 40 (3.8) | 0.043 |
| Renal Failure | 8 (1.3) | 69 (1.8) | 0.040 | 8 (1.4) | 9 (0.8) | 0.053 |
| Liver Disease | 15 (2.5) | 136 (3.7) | 0.065 | 14 (2.4) | 28 (2.6) | 0.011 |
| Peptic Ulcer Disease excluding bleeding | 2 (0.3) | 18 (0.4) | 0.023 | 2 (0.3) | 5 (0.4) | 0.019 |
| AIDS/HIV | 4 (0.6) | 36 (0.9) | 0.032 | 4 (0.7) | 9 (0.8) | 0.017 |
| Lymphoma | 0 (0.0) | 0 (0.0) | 0.000 | 0 (0.0) | 0 (0.0) | 0.000 |
| Metastatic Cancer | 0 (0.0) | 0 (0.0) | 0.000 | 0 (0.0) | 0 (0.0) | 0.000 |
| Solid Tumor without Metastasis | 0 (0.0) | 0 (0.0) | 0.000 | 0 (0.0) | 0 (0.0) | 0.000 |
| Rheumatoid Arthritis/collagen | 1 (0.1) | 37 (1.0) | 0.109 | 1 (0.1) | 1 (0.0) | 0.022 |
| Coagulopathy | 9 (1.5) | 40 (1.0) | 0.040 | 9 (1.6) | 5 (0.4) | 0.111 |
| Obesity | 60 (10.3) | 423 (11.5) | 0.039 | 57 (10.1) | 94 (8.9) | 0.040 |
| Weight Loss | 8 (1.3) | 28 (0.7) | 0.060 | 7 (1.2) | 3 (0.2) | 0.110 |
| Fluid and Electrolyte Disorders | 31 (5.3) | 302 (8.2) | 0.116 | 29 (5.1) | 48 (4.5) | 0.027 |
| Blood Loss Anemia | 1 (0.1) | 15 (0.4) | 0.044 | 1 (0.1) | 3 (0.2) | 0.022 |
| Deficiency Anemia | 17 (2.9) | 122 (3.3) | 0.023 | 14 (2.4) | 24 (2.2) | 0.013 |
| Alcohol Abuse | 74 (12.7) | 568 (15.4) | 0.079 | 71 (12.6) | 135 (12.8) | 0.007 |
| Drug Abuse | 151 (25.9) | 1,201 (32.7) | 0.149 | 147 (26.1) | 301 (28.6) | 0.057 |
| Psychoses | 582 (100.0) | 3,670 (100.0) | 0.000 | 562 (100.0) | 1,049 (100.0) | 0.000 |
| Depression | 108 (18.5) | 987 (26.8) | 0.200 | 101 (17.9) | 170 (16.2) | 0.047 |

CCI, Charlson Comorbidity Index; PP1M, paliperidone palmitate 1-monthly; PP3M, paliperidone palmitate 3-monthly; PSM, propensity score matching; SMD, standardized mean difference; SD, standard deviation

**Supplementary Table 7.** **Additional analysis-** proportion of patients who had relapse and relapse criteria based on PSM (1:2)

| **Relapse definition** | **Proportion of patients who had relapse** | | **Proportion of patients who had relapse per Japan label** | | |
| --- | --- | --- | --- | --- | --- |
|  | **PP3M, n (%)** | **PP1M, n (%)** | **PP3M, n (%)** | | **PP1M, n (%)** |
| **Number accessed** | 562 | 1049 | 562 | | 1049 |
| **Number of censored, n (%)** | 448 (79.7) | 772 (73.6) | 508 (90.4) | | 916 (87.3) |
| **Number of relapse, n (%)** | 114 (20.3) | 277 (26.4) | 54 (9.6) | | 133 (12.7) |
| **Relapse-free status*, %** | 78.4 | 72.2 | 85.7 | | 79.9 |
| **Difference (PP3M-PP1M)** | 6.2 | | 5.8 | | |
| **95% CI^†^** | (1.7; 10.7) | | (0.8; 10.9) | | |
| **Reason of relapse, n (%)** | | | | | |
| Violent behavior resulted in suicide | 22 (3.9) | 51 (4.9) | 14 (2.5) | 29 (2.8) | |
| Suicidal ideation | 5 (0.9) | 18 (1.7) | 3 (0.5) | 8 (0.8) | |
| Homicidal ideation | 3 (0.5) | 5 (0.5) | 1 (0.2) | 3 (0.3) | |
| Suicidal and Homicidal ideation | 1 (0.2) | 2 (0.2) | 1 (0.2) | 0 (0.0) | |
| Deliberate self-injection/Violent behavior | 3 (0.5) | 4 (0.4) | 0 (0.0) | 3 (0.3) | |
| Other^‡^ | 1 (0.2) | 6 (0.6) | 0 (0.0) | 4 (0.4) | |
| Inpatient psychiatric hospitalization (IPH) alone | 50 (8.9) | 101 (9.6) | 25 (4.4) | 49 (4.7) | |
| IPH + Homicidal ideation | 4 (0.7) | 13 (1.2) | 0 (0.0) | 7 (0.7) | |
| IPH + Deliberate self-injection/Violent behavior | 3 (0.5) | 2 (0.2) | 0 (0.0) | 0 (0.0) | |
| IPH + Violent behavior resulted in suicide | 3 (0.5) | 19 (1.2) | 0 (0.0) | 7 (0.7) | |
| IPH + Suicidal and Homicidal ideation | 1 (0.2) | 3 (0.3) | 1 (0.2) | 3 (0.3) | |
| IPH + Suicidal ideation | 18 (3.2) | 53 (5.1) | 9 (1.6) | 20 (1.9) | |

^*^Kaplan-Meier estimate

^†^Non-inferiority of PP3M to PP1M will be concluded if the lower limit of the 2-sided 95% CI of the difference proportion patients who were in relapse-free between PP3M and PP1M exceeded the pre-selected non-inferiority margin of -10%. PP3M will be declared superior to PP1M if the lower limit of the 2-sided 95% CI of the difference in the proportion of patients who were relapse-free between PP3M and PP1M exceeded 0%.

^‡^Suicidal ideation, deliberate self-injection/violent behavior, violent behavior resulted in suicide.

CI, confidence interval; IPH, inpatient psychiatric hospitalization; PP1M, paliperidone palmitate 1-monthly; PP3M, paliperidone palmitate 3-monthly; PSM, propensity score matching.

**Supplementary Figure 1. Additional analysis-** PP3M and PP1M propensity score distribution before and after PSM (1:2)


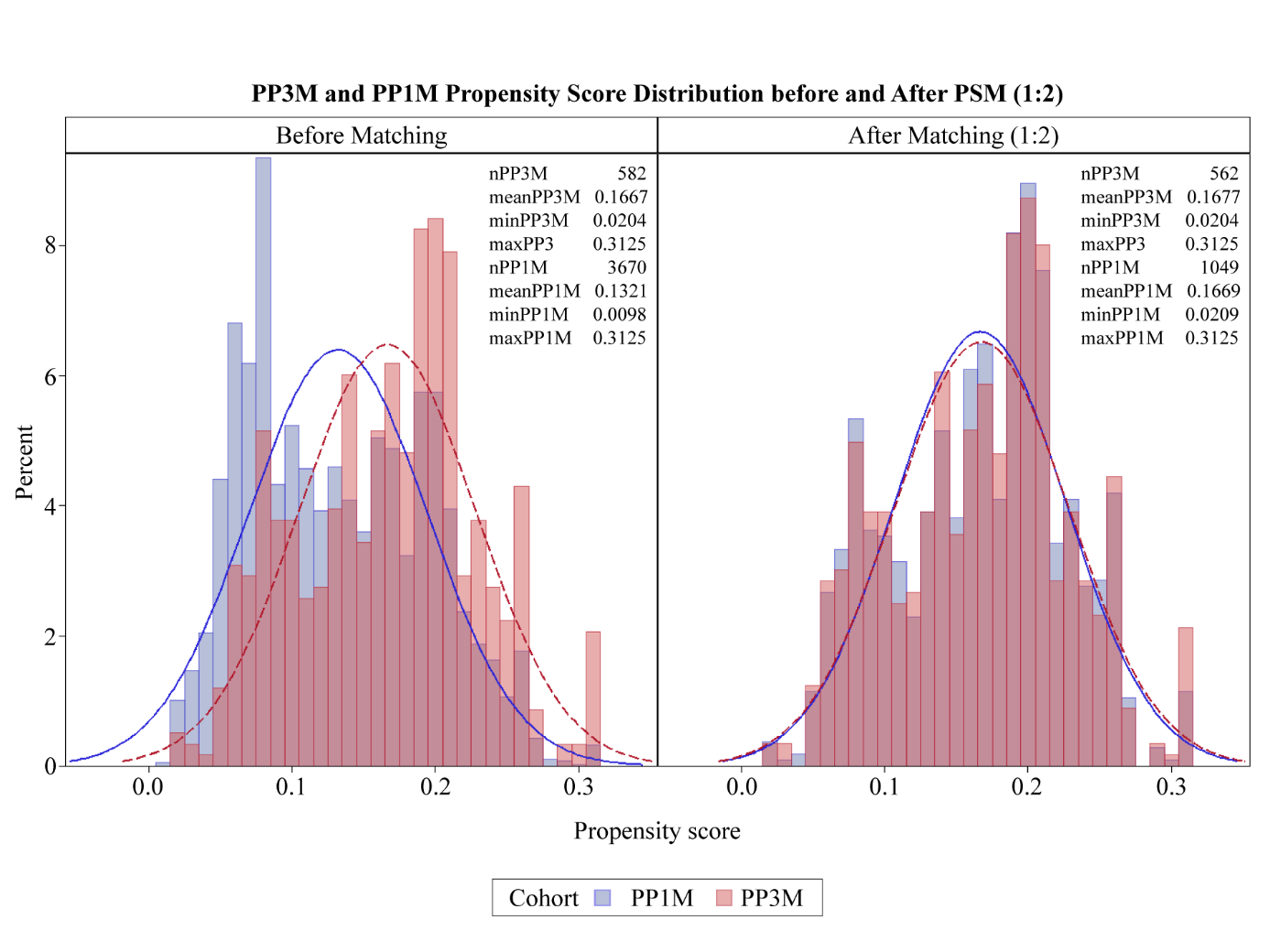


PP1M, paliperidone palmitate 1-monthly; PP3M, paliperidone palmitate 3-monthly; PSM, propensity score matching

**Supplementary Figure 2. Additional analysis-** proportion of patients relapse free and time to relapse (PSM 1:2): (A) Time to first relapse and proportion of patients relapse-free (B) Time to first relapse and proportion of patients relapse-free based on Japan label


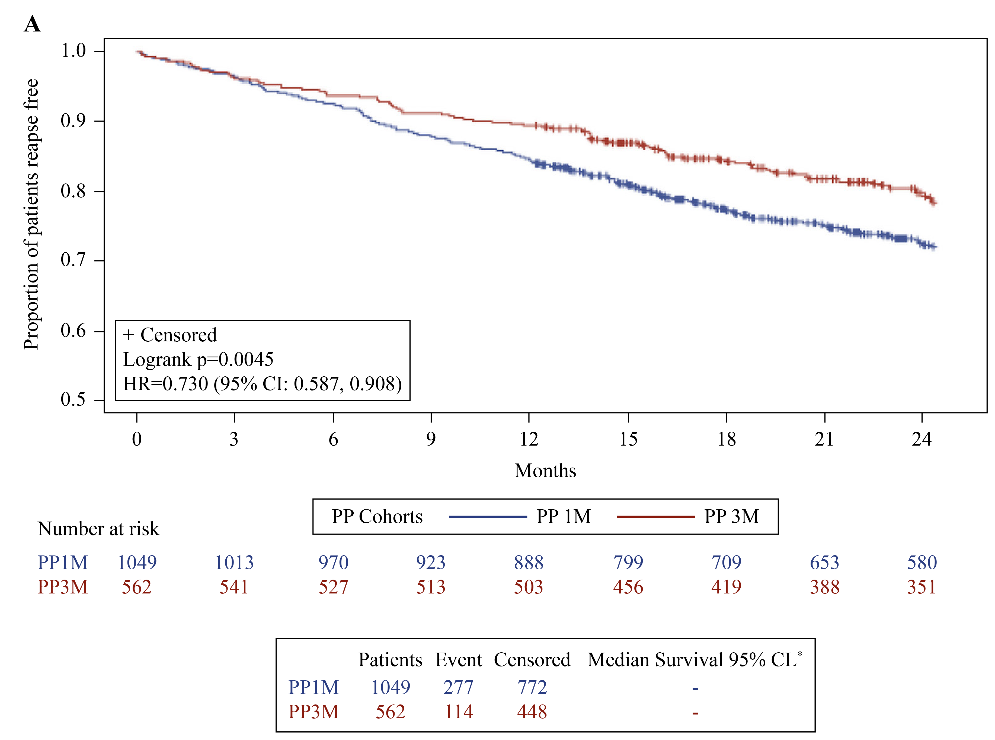


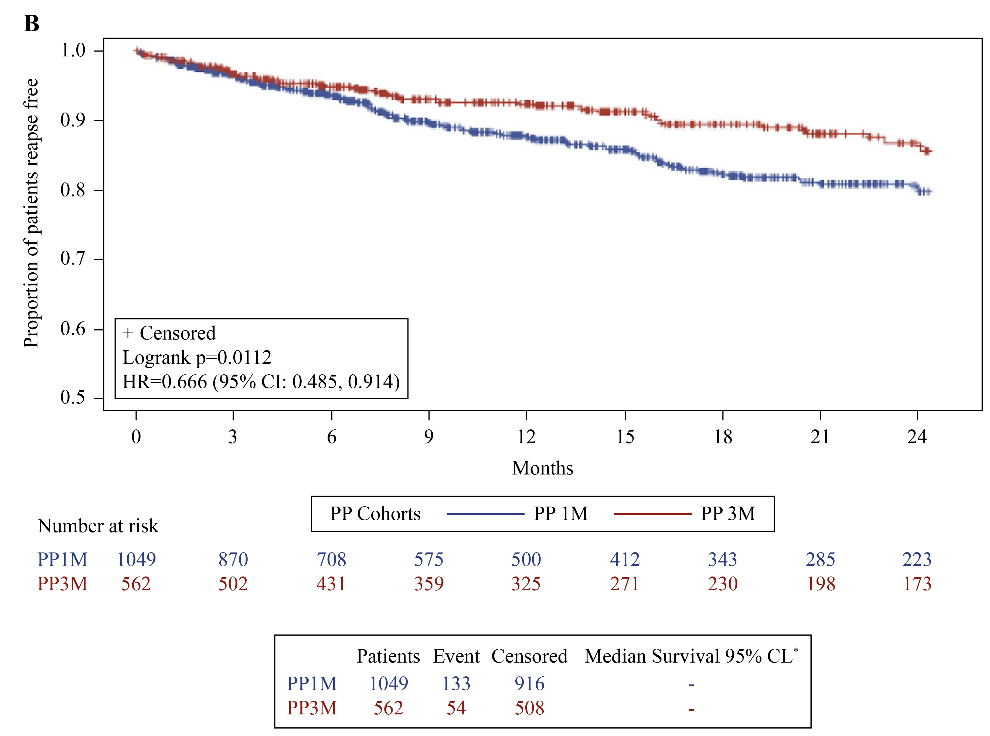


*Median time to relapses could not be calculated as 50% of patients did not have a relapse.

CI, confidence interval; HR, hazard ratio; PP1M, paliperidone palmitate 1-monthly; PP3M, paliperidone palmitate 3-monthly; PSM, propensity score matching.

**Supplementary Figure 3. Additional analysis**- treatment adherence (PSM 1:2)**:** (A) Distribution of cohorts (%) by PP1M/PP3M adherence categories (B) Distribution of relapse rates by PP1M/PP3M adherence categories


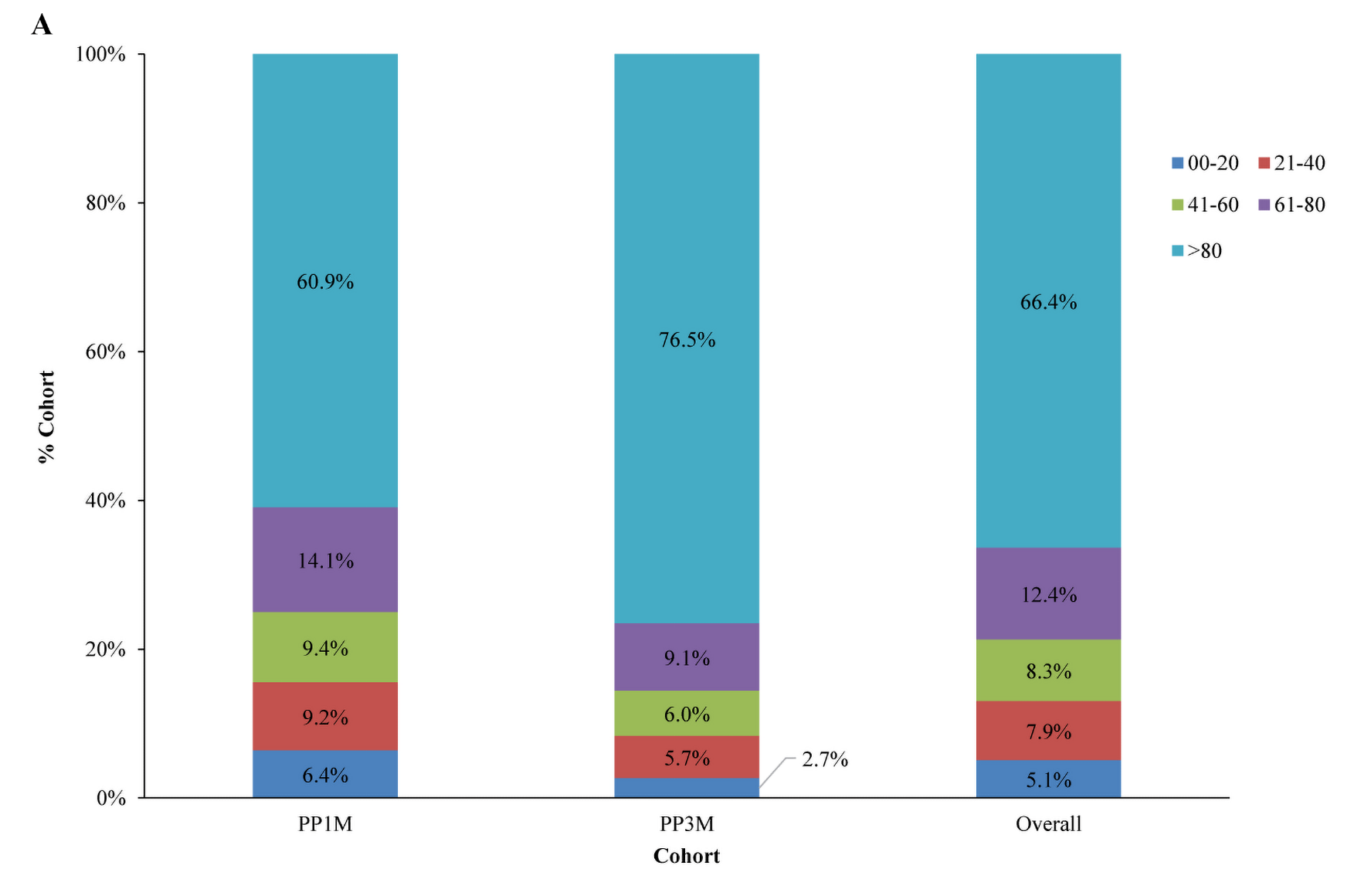


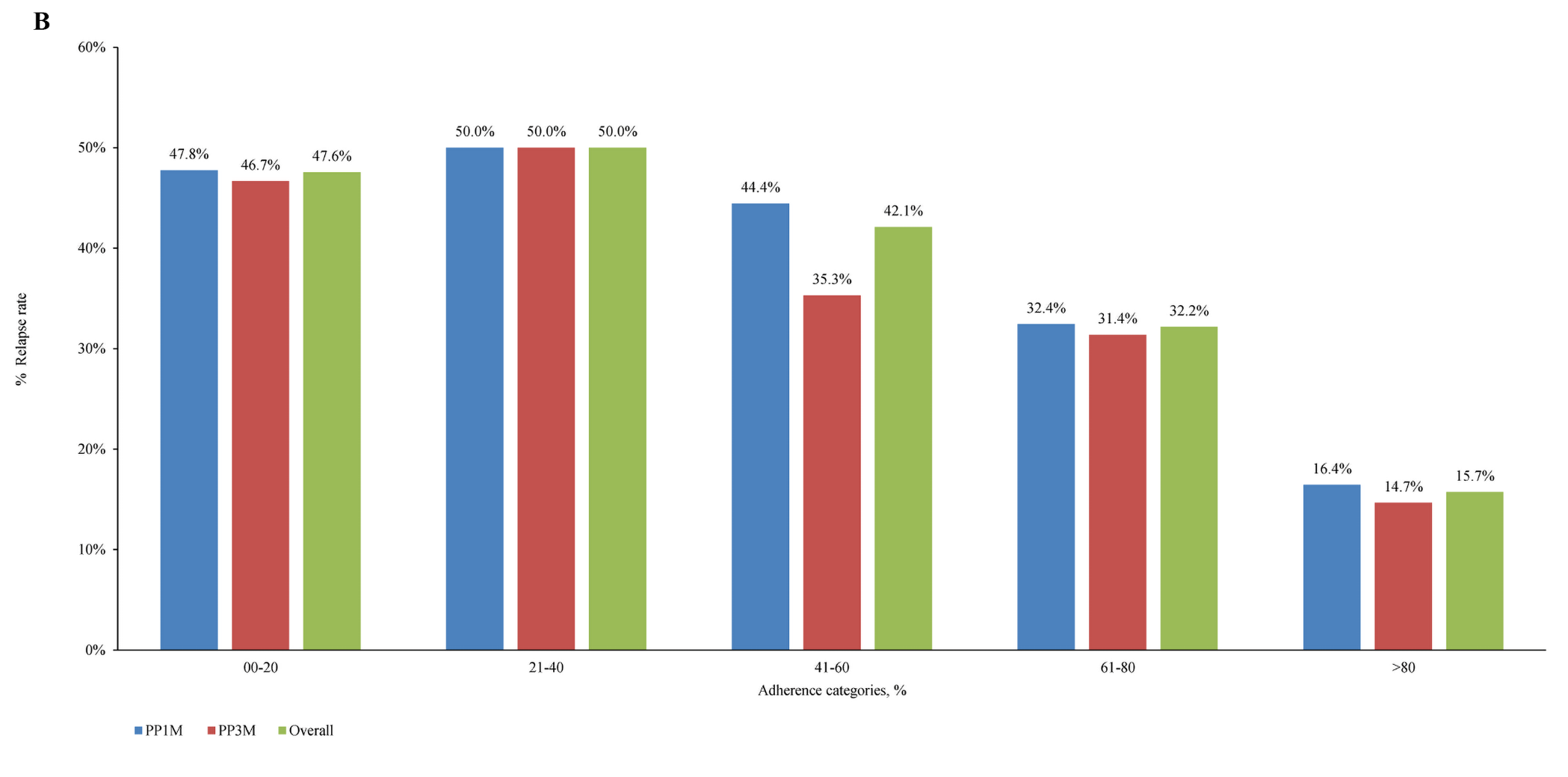


PP1M, paliperidone palmitate 1-monthly; PP3M, paliperidone palmitate 3-monthly
